# Comparative proteomic analysis of normal and gliotic PVR retina and contribution of Müller glia to this profile

**DOI:** 10.1016/j.exer.2018.08.016

**Published:** 2018-12

**Authors:** Karen Eastlake, Wendy E. Heywood, Phillip Banerjee, Emily Bliss, Kevin Mills, Peng T. Khaw, David Charteris, G. Astrid Limb

**Affiliations:** a NIHR Biomedical Research Centre at Moorfields Eye Hospital and UCL Institute of Ophthalmology, London, UK; b Centre for Translational Omics, UCL Great Ormond Street Institute of Child Health, London, UK

**Keywords:** Müller glia, Proteomics, Retinal gliosis, Retina degeneration

## Abstract

Müller glia are responsible for the neural retina regeneration observed in fish and amphibians throughout life. Despite the presence of these cells in the adult human retina, there is no evidence of regeneration occurring in humans following disease or injury. It may be possible that factors present in the degenerated retina could prevent human Müller glia from proliferating and neurally differentiating within the diseased retina. On this basis, investigations into the proteomic profile of these cells and the abundance of key proteins associated to Müller glia in the gliotic PVR retina, may assist in the identification of factors with the potential to control Müller proliferation and neural differentiation *in vivo*. Label free mass spectrometry identified 1527 proteins in Müller glial cell preparations, 1631 proteins in normal retina and 1074 in gliotic PVR retina. Compared to normal retina, 28 proteins were upregulated and 196 proteins downregulated by 2-fold or more in the gliotic PVR retina. As determined by comparative proteomic analyses, of the proteins highly upregulated in the gliotic PVR retina, the most highly abundant proteins in Müller cell lysates included vimentin, GFAP, polyubiquitin and HSP90a. The observations that proteins highly upregulated in the gliotic retina constitute major proteins expressed by Müller glia provide the basis for further studies into mechanisms that regulate their production. In addition investigations aimed at controlling the expression of these proteins may aid in the identification of factors that could potentially promote endogenous regeneration of the adult human retina after disease or injury.

## Highlights

• Proteomic analyses showed evidence for Müller glia contribution to retinal gliosis.
• Polyubiquitin-C and HSP90a produced by Müller glia, are upregulated in gliotic retina.
• Müller glia are a source of prelamin, elongation factor and serpin found in retina.

## 1 Introduction

A common characteristic of retinal degeneration is reactive gliosis, a term coined to describe a process where Müller glia rapidly proliferate (Dyer and Cepko, 2000), undergo morphological changes (Lewis and Fisher, 2003), and release pro-inflammatory and regulatory factors into the local retinal environment (Bringmann et al., 2009). This process, thought to protect the retina from further damage, is not always beneficial and can lead to the formation of glial scarring which further aggravates retinal degeneration (Bringmann and Wiedemann, 2012). As Müller cell gliosis is the main characteristic of retinal degenerative disorders (Fariss et al., 2000; Graf et al., 1993; Rungger-Brandle et al., 2000; Wu et al., 2003), a greater understanding of the molecular events that occur in this process is required to develop therapeutic strategies to promote self-repair and consequently endogenous regeneration.

Müller glia provide structural and homeostatic support to retinal neurons and constitute the first line of reactivity upon retinal damage. Whilst Müller glia are responsible for the regeneration observed in zebrafish retina after injury, there is no evidence that these cells have regenerative ability in humans. On the contrary, Müller cells are known to undergo changes which lead to reactive gliosis and scarring of the human retina (Bringmann and Wiedemann, 2012). A population of Müller glia with stem cell characteristics (hMSC) has been identified in the adult human retina (Bhatia et al., 2009b; Lawrence et al., 2007). Upon isolation *in vitro* these cells become spontaneously immortalized (Limb et al., 2002) and can be induced to express markers of retinal ganglion cells or photoreceptor neurons by culture with growth and differentiation factors (Jayaram et al., 2014; Singhal et al., 2012). In addition, Müller glia can be induced to proliferate *in situ* in human retinal explants cultured with growth and differentiation factors (Bhatia et al., 2009a). Although there is no evidence that Müller glia proliferate or neurally differentiate *in situ*, the observations that they can proliferate indefinitely *in vitro* and differentiate into retinal neurons upon culture with differentiation factors, suggest that Müller glia may have the potential to repair the human retina *in vivo.* However for unknown developmental reasons, they are prevented from exerting this regenerative function.

Various studies have examined the proteome profile of the retina using experimental disease models (Böhm et al., 2013; Kim et al., 2012; Ly et al., 2014; Tu et al., 2013), but proteomic investigations of the gliotic human retina have been limited. Recent studies however, have identified 3436 non-redundant proteins in the normal human retina (Zhang et al., 2015), contributing to the mapping of the human retinal proteome which may aid investigations into disease mechanisms. Several proteomic studies on the characterisation of Müller glia has been performed on cells isolated from rodent or porcine retina (Grosche et al., 2015; Hauck et al., 2003; Merl et al., 2012), but investigations into the proteomic profile of human Müller glia in the context of retinal gliosis have not been reported. This study has therefore attempted to examine the contribution of human Müller glia to the proteomic profile of the gliotic human retina by comparing the protein expression profiles of gliotic and normal human retina with that of Müller glia cell lines *in vitro*.

## 2 Materials and methods

### 2.1 Tissue acquisition

Retinae from four normal cadaveric donors with prior consent for research were obtained from Moorfields eye Bank under 24 h post mortem. Specimens for protein analysis were obtained by excising sections of normal peripheral retina between 1 and 3 mm x 1–5 mm (3–5 mm^2^) to match the size of the retinectomy specimens obtained. Six peripheral retinectomy specimens (3–5 mm^2^) from eyes undergoing retinal surgery for treatment of proliferative vitreo-retinopathy (PVR) (duration of 2–10 weeks) were obtained according to guidelines from the Local Ethics Committee at Moorfields and the Institute of Ophthalmology and followed the tenets of the Declaration of Helsinki. Donors ages ranged from 32 to 83yrs and matched where possible. Before pooling for the proteomics assays, specimens were washed in phosphate buffered saline (PBS) and frozen at −80 °C until use.

### 2.2 Cell culture

The immortalized human Müller cell line MIO-M1 (Limb et al., 2002) was used in this study along with the immortalized Müller cell preparations 6387 (MIO-M7), 6391(MIO-M4) and 6390 (MIO-M5) generated in our laboratory. Cells were grown to confluence in 10% foetal calf serum (FCS, Invitrogen, UK) in DMEM with GlutaMAX (31966-021; Invitrogen UK) at 37 °C in the presence of 5% CO_2_.

### 2.3 Protein isolation and proteomic analysis of human retina and Müller glial cells

Retinal fragments and Müller cell preparations were washed in PBS prior to homogenisation with 50 nM Ammonium Bicarbonate +2%ASB-14 (Sigma Aldrich, UK) pH8.2. Protein content was estimated using the Bicinchoninic Acid (BCA) protein assay kit (Sigma-Aldrich, UK).

### 2.4 2-Dimensional gel electrophoresis (2D-DIGE) assay

Human retina protein isolated from 4 normal, 5 gliotic specimens, as well as 4 Müller glia cell preparations were combined to create 3 separate pools, each consisting of 150 μg of protein. 2D-DIGE analyses were performed as previously described (Heywood et al., 2011). Each protein pool was labelled with a different CyDye (600 pmol) as follows: Control retina was labelled with Cy3; gliotic retina with Cy5 and Müller glia with Cy2. Samples were then combined and added to Immobiline DryStrip gels (IPG) and run on an IPG Multiphor II Electrophoresis System (GE Healthcare, Little Chalfont, U.K.). For the second dimension, Isoelectric focusing (IEF) strips were re-equilibrated before resolving samples on an Ettan DALT twelve System separation tank using 12% acrylamide gels. Gels were fixed and imaged using a Typhoon scanner (Model 8600, GE Healthcare, UK). Spot comparison was performed using Progenesis Samespots software (Non-Linear Dynamics, Waters, UK). Gels were silver stained (Heywood et al., 2011) and protein spots excised, trypsin digested and processed as for mass spectrometry.

### 2.5 Label free proteomics

Similarly, three different pools from normal, gliotic human retina and Müller glia were used for label free proteomics. Freeze-dried samples (80 μg each) were reconstituted in homogenisation buffer (50 mM AmmoniumBicarb + 2%ASB-14) and run on a 1D gel (BioRad Mini Protean Precast gels, BioRad, UK) as described previously (Heywood et al., 2011). Ten gel bands of each sample lane were excised according to molecular weight and subjected to trypsin digestion and peptide extraction as previously described (Bennett et al., 2010). Digests were reconstituted in 30 μl of 3%ACN +0.1% Trifluoroacetic acid (TFA) with yeast enolase added as an internal standard and analysed using a QToF Premier™ mass spectrometer coupled to a NanoAquity Nano-LC system (Waters Corp, UK).

### 2.6 Data analysis

Specimens (normal retina, gliotic retina and Müller glia) were assessed for differentially expressed proteins giving an n number of 1 for each condition. Therefore, the high cut-off values of ≥2-fold upregulated or ≤0.5-fold downregulated were chosen to obtain higher confidence when analysing the data.

Proteins were identified using ProteinLynx Global Server (PLGS) 2.4 (Waters, UK) with a downloaded Uniprot reference proteome for homo sapiens with porcine trypsin (Accession: P00761) and yeast enolase (Accession: P00924) added. Results generated were further processed through Progenesis QI software (Non-linear dynamics, Waters UK). Only proteins identified by 2 or more peptides were used in comparative analyses, Supplementary Table 1 contains the list of proteins used in the analysis. Further analyses of the results were conducted on web-based platforms including the Panther Classification System (http://www.pantherdb.org/), (Mi et al., 2013) gene ontology classification by WebGestalt (http://bioinfo.vanderbilt.edu/webgestalt/) (Wang et al., 2013) and IMPaLA (http://impala.molgen.mpg.de/) for pathway analysis (Kamburov et al., 2011).

Mass spectrometry proteomics data have been deposited in the ProteomeXchange Consortium database (http://proteomecentral.proteomexchange.org) via the PRIDE partner repository (Vizcaino et al., 2013) with the dataset identifier PXD008485.

### 2.7 Western blot analysis

Cell lysis and western blot analysis was performed as previously described (Lawrence et al., 2007). Aliquots of cell lysates were resolved on 4–12% NuPAGE Bis-Tris gels (Invitrogen, U.K.). Proteins were transferred to PDVF membranes (Semi-dry transfer, Biorad,UK) and blocked with 5% FBS and 5% skimmed milk powder in TBS. Immunodetection was performed using primary antibodies against vimentin (1:250; Santa Cruz), galectin-1 (1:5000; Abcam), and GFAP (1:2000; DAKO). Protein bands were detected following incubation with donkey antiserum against rabbit, or mouse IgG coupled to horseradish peroxidase (Santa Cruz Biotechnology Inc). Chemiluminescence was detected using Millipore Luminata detection reagents and resolved using an x-ray developer.

### 2.8 Immunohistochemistry

Human tissue samples from normal retina or gliotic PVR retina were fixed in 4% PFA for 30mins, cryopreserved in 30% sucrose and embedded in OCT for cryosectioning. For immunostaining, sections were blocked (TBS + 0.3% triton X 5% donkey serum) for 1 h prior to the addition of the primary antibody, incubated overnight at 4 ° C. Sections were then washed with TBS and incubated in secondary antibodies (Alexa flour, 1:500 in TBS + 0.3% triton) for 3 hrss at room temperature in the dark. Slides were then washed in TBS and counterstained with a mounting medium containing DAPI (Fluoroshield; Abcam) before coverslipping.

## 3 Results

### 3.1 Proteomic profiles of Müller glial cell lines and of normal and gliotic human retina examined by label free analysis

**Fig. 1:**
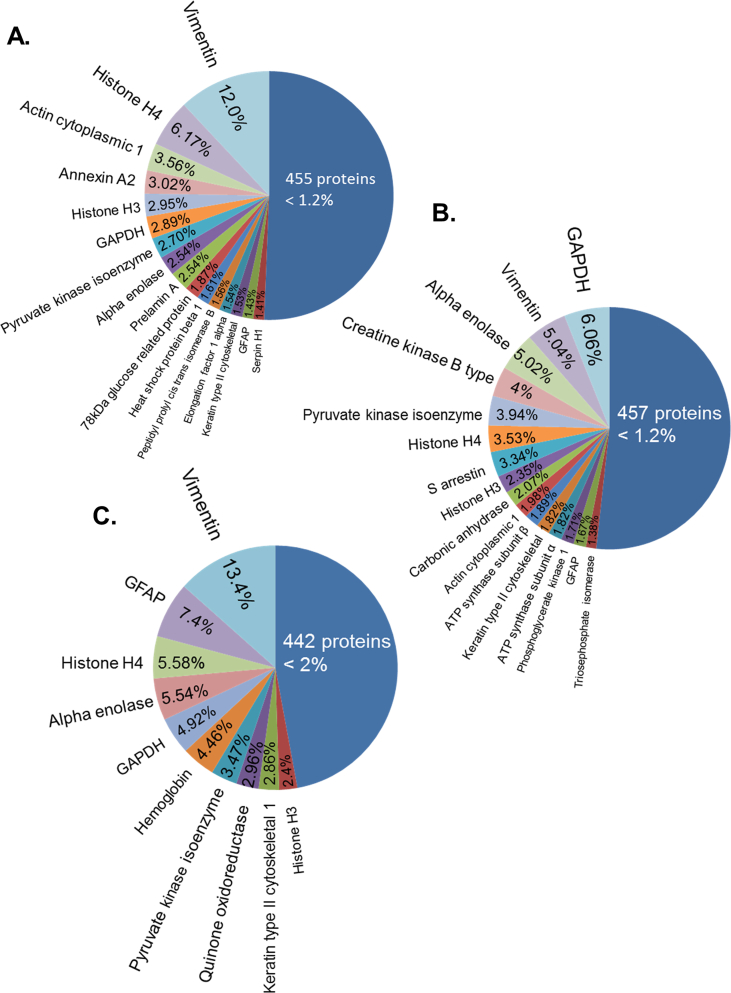
**Abundance of proteins as identified by label free mass spectrometry analysis in normal retina, gliotic retina and Müller glia**. For clarity only proteins with the highest abundance are labelled. (A) Pie chart shows protein abundance represented as percentage of total protein identified in a pool of Müller glial cell lysate. (B) Pie chart shows protein abundance represented as percentage of total protein identified in a pool of 4 normal retinae. (C) Pie chart shows protein abundance represented as percentage of total protein identified in a pool of 5 gliotic retinae.

Analysis of protein expression by label free mass spectrometry identified 1527 proteins in Müller glial cell preparations, 1631 proteins in normal retina and 1074 in gliotic PVR retina. In Müller glial cell preparations vimentin exhibited the highest abundancy (14% of total protein), but GFAP only constituted 1.43% of the protein content. Other proteins highly expressed in Müller glia included histone H4 (6.17%), actin cytoplasmic 1 (3.56%) and annexin A2 (3.02%) (Fig. 1A). In normal retina GAPDH was the most abundant protein and constituted 6.06% of the total protein. This was followed by vimentin (5.04%), alpha enolase (5.02%) and creatine kinase b type (4%) (Fig. 1B). In gliotic retina, vimentin was the most abundant protein, constituting 13.4% of total protein, followed by GFAP (7.4%), histone H4 (5.58%) and alpha enolase (5.54%) (Fig. 1C). Supplementary Table 1 shows the full list of identified proteins in each group which were used for analysis.

### 3.2 Differences in protein expression between normal and gliotic PVR retina and Müller glial profile

**Fig. 2:**
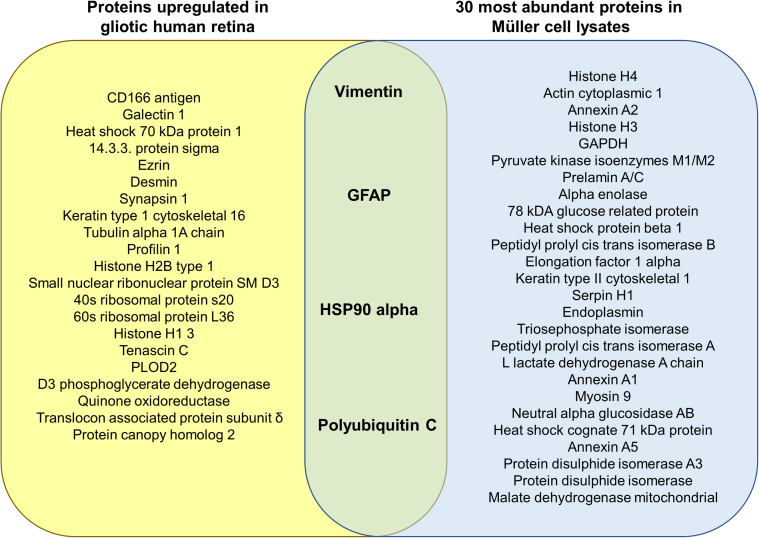
**Proteins upregulated in the gliotic retina are among the 30 most abundant proteins expressed by Muller glia**. Venn diagram shows the proteins upregulated in the gliotic human retina (yellow area) and the 30 most abundant proteins in the Müller glia cell preparation (blue area) and the common proteins to both groups (green area). (For interpretation of the references to colour in this figure legend, the reader is referred to the Web version of this article.)

As identified by label free mass spectrometry and based on a >2-fold increase in expression, 28 proteins were upregulated in the gliotic retina as compared to normal retina. Quinone oxidoreductase exhibited the highest fold change (9.22-fold) followed by CD166 (8.2-fold) and protein canopy homolog 2 (6.83-fold). A large proportion of upregulated proteins are involved in cytoskeletal regulation and included the filament proteins ezrin, glial fibrillary acidic protein, desmin and vimentin. Nucleic acid binding proteins were also upregulated in gliotic retina and included histones, ribosomal proteins and poly-ubiquitin (Table 1). Interestingly, all the proteins found upregulated in the gliotic retina, were also present in the Müller cell preparation. These have been highlighted in Table 1, showing the associated ranked abundancies within the Müller cell preparation. Of the 30 most abundant proteins identified by mass spectrometry in Müller glial cell lysates, vimentin showed the highest abundance, followed by histone H4 and actin cytoplasmic 1 (Fig. 2). Four of the 30 most abundant proteins observed in Müller cell preparations were found highly upregulated in the gliotic retina as compared to normal retina. These included vimentin, GFAP, heat shock protein 90α and polyubiquitin C (Fig. 2).

**Table 1:** **Proteins upregulated** > **2-fold in gliotic human retina compared to normal retina.** Table shows the proteins found upregulated >2-fold in the gliotic retina as compared to normal retina. They have been grouped according to their major functions for clarity. Ranked abundancies for each protein found in the Müller cell preparation are listed (Low numbers indicate higher abundancy, e.g. 1 = highest abundant protein in the Müller cell preparation).

Protein Class	Upregulated				Ranked Abundance in Müller preparation
	Protein	Accession	No. Peptides	Fold change	
Cell adhesion molecule	CD166 antigen	Q13740	3	8.20	274
	Galectin 1	P09382	25	2.03	56
Chaperone	Heat shock protein HSP90 alpha	P07900	76	2.0	23
	Heat shock 70 kDa protein 1	P34931	32	6.70	396
	14 3 3 protein sigma	P31947	21	4.31	216
Structural molecule activity	Ezrin	P15311	20	5.25	280
	Glial fibrillary acidic protein	P14136	217	4.50	15
	Desmin	P17661	56	4.84	154
	Synapsin 1	P17600	2	5.39	369
	Keratin type 1 cytoskeletal 16	P08779	18	3.37	358
	Tubulin alpha 1A chain	Q71U36	199	2.64	86
	Vimentin	P08670	432	2.53	1
	Profilin 1	P07737	21	2.01	36
Membrane trafficking protein	Synapsin 1	P17600	2	5.39	369
Nucleic acid binding	Histone H2B type 1	P23527	143	3.80	289
	Small nuclear ribonucleoprotein SM D3	P62318	4	2.83	178
	Polyubiquitin 3	P0CG48	37	2.06	28
	40s ribosomal protein s20	P60866	2	2.32	272
	60s ribosomal protein L36	Q9Y3U8	5	3.90	53
	Histone H1 3	P16402	23	2.01	81
Signalling molecule	Galectin 1	P09382	25	2.02	56
	Tenascin C	P24821	2	2.54	188
Oxioreductase	Procollagen lysine 2 oxoglutarate 5 dioxygenase 2	O00469	6	2.31	158
	D3 phosphoglycerate dehydrogenase	O43175	3	2.13	375
	Quinone oxidoreductase	Q08257	4	9.22	75
MISC	Translocon associated protein subunit delta	P51571	4	2.17	256
	Protein canopy homolog 2	Q9Y2B0	2	6.83	354

Well known Müller glia markers were also predominant in Müller cell preparations and included glutamine synthetase, retinaldehyde binding protein 1 and CD44-antigen. Of these, both glutamine synthetase and retinaldehyde binding protein 1 were downregulated in the gliotic retina as compared to normal retina. Several proteins present in Müller cell lysates have not been previously identified in Müller glia and included prelamin A/C, elongation factor 1, and serpin H1. Further details can be found in Supplementary Table 2.

Based on <0.5-fold decrease in expression, 196 proteins were downregulated in the gliotic retina as compared to normal retina. Mitochondrial 2 oxoglutarate malate carrier protein showed the greatest decrease in the gliotic retina (0.005-fold), followed by Tubulin alpha 8 chain (0.01-fold) and reticulon 4 (0.1-fold). The majority of downregulated proteins identified are involved in nucleic acid binding including ribosomal related proteins (Table 2).

**Table 2:** **Proteins downregulated in the gliotic human retina compared to normal retina.** Table shows the 50 proteins found to be downregulated <0.5-fold in the gliotic retina as compared to normal retina. They have been grouped according to their major functions for clarity.

Protein Class	Downregulated			
	Protein	Accession	No.Peptides	Fold change
Chaperone	Putative Heat shock protein 90 beta 2	Q58FF8	35	0.04

Structural molecule activity	Keratin type II cuticular Hb3	P78385	11	0.06
	Tubulin alpha 8 chain	Q9NY65	83	0.01
	Keratin type II cuticular Hb2	Q9NSB4	3	0.08
	Neurofilament medium polypeptide	P07197	16	0.06
	Tropomyosin alpha 1 chain	P09493	18	0.07
Membrane trafficking protein	Reticulon 4	Q9NQC3	2	0.01
	Endoplasmic reticulum resident protein 29	P30040	5	0.15
	Coatomer subunit alpha	P53621	4	0.12
Nucleic acid binding	Matrin 3	P43243	3	0.09
	Proliferating cell nuclear antigen	P12004	2	0.04
	60S ribosomal protein L24	P83731	3	0.04
	Poly rC binding protein 3	P57721	7	0.03
	Poly ADP ribose polymerase 1	P09874	2	0.02
	40S ribosomal protein S11	P62280	7	0.11
	Transcriptional activator protein Pur alpha	Q00577	2	0.08
	ADP ATP translocase 1	P12235	46	0.07
	40S ribosomal protein S4 X	P62701	6	0.02
	60S ribosomal protein L13a	P40429	2	0.13
	40S ribosomal protein S9	P46781	8	0.09
	Core histone macro H2A 1	O75367	13	0.13
	60S ribosomal protein L13	P26373	4	0.02
	Mitochondrial 2 oxoglutarate malate carrier protein	Q02978	6	0.005
	40S ribosomal protein S26	P62854	3	0.12
	60s Ribosomal protein L14	P50914	6	0.11
	Non histone chromosoaml protein HMG	P05114	5	0.14
	40S ribosomal protein S23	P62266	2	0.13
	Polyadenylate binding protein 1	Q4VXU2	2	0.13
	ATP synthase subunit gamma mitochondrial	P36542	9	0.14
	Guanine nucleotide binding protein GIGSGT subunit beta 1	P62873	29	1.13
	Adenylate kinase isoenzyme 1	P00568	2	0.14
Signalling molecule	Membrane associated progesterone receptor component 1	O00264	4	0.12
Transferase	Hypoxanthine guanine phosphoribosyltransferase	P00492	4	0.03
	Creatine kinase U type mitochondrial	P12532	4	0.07
	ATP citrate synthase	P53396	4	0.12
	Guanylate kinase	Q16774	4	0.09
Oxioreductase	Peroxiredoxin 4	Q13162	10	0.06
	NADH cytochrome b5 reductase 3	P00387	6	0.08
	Estradiol 17 beta dehydrogenase 12	Q53GQ0	2	0.05
	Glycerol 3 phosphate dehydrogenase mitochondrial	P43304	3	0.07
	Isocitrate dehydrogenase NAD subunit alpha	P50213	4	0.14
	Procollagen lysine 2 oxoglutarate 5 dioxygenase 3	O60568	7	0.15
Detoxification	Glutathione S-transferase Mu 2	P28161	4	0.02
Enzymes/modulators	Interleukin enhancer binding factor 3	Q129606	5	0.14
	Inorganic pyrophosphatase	Q15181	3	0.02
	Poly rC binding protein 2	Q15366	8	0.14
Transcription factor	Cullin associated NEDD8 dissociated protein 1	Q86VP6	3	0.10
	Transcriptional activator protein Pur alpha	Q00577	2	0.08
MISC	Proteasome subunit alpha type 7	O14818	2	0.06
	Prohibitin	P35232	8	0.13
	Thioredoxin domain containing protein 5	Q8NBS9	2	0.13

### 3.3 Comparative analysis of protein expression between Müller glia and normal and gliotic PVR retina as examined by 2D-DIGE

**Fig. 3:**
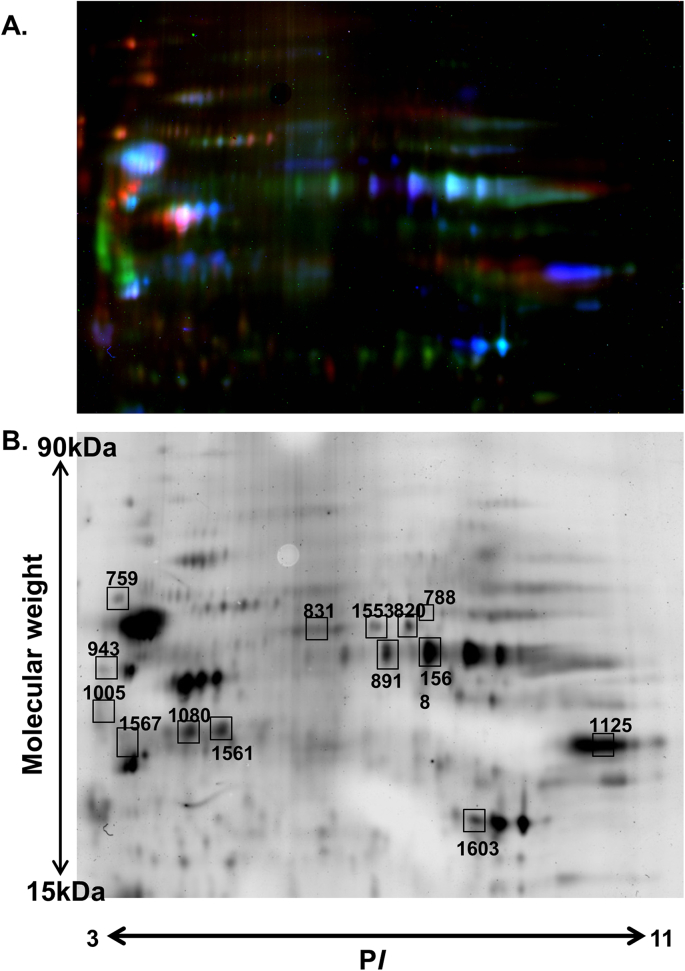
**2D-DIGE gel analysis of normal and gliotic human retina.** (A) Fluorescent gel image showing labelled proteins from a pool of 4 Müller glial cell preparations (Cy2 = green), 5 gliotic retinal specimens (Cy5 = Blue) and 4 normal retinae specimens (Cy3 = Red). (B) Representative 2D DIGE gel of a pool of 4 normal retina. Numbered protein spots are those shown to be differentially expressed between the gliotic and human retina by more than 2-fold. Table 3 shows the protein identification of the numbered spots shown in this Figure. (For interpretation of the references to colour in this figure legend, the reader is referred to the Web version of this article.)

Approximately 200 protein spots were detected in the 2D-DIGE analysis. Based on a >2-fold increase in intensity, 8 spots showed >2-fold upregulation in the gliotic as compared to normal retina, whilst 23 protein spots showed a <0.5-fold decrease. In addition, all proteins observed differentially expressed were present in the Müller cell lysates (Fig. 3A and B). Proteins from 14 representative gel spots were extracted and digested prior to mass spectrometry to identify individual proteins. For each spot, the protein with the highest peptide count was considered the protein of interest. Proteins showing upregulation in the gliotic retina as compared to normal retina supported the current mass spectrometry observations that GFAP, vimentin and aminopeptidase are highly upregulated in during gliosis. Proteins identified to be downregulated in the gliotic retina as compared to normal retina by 2D-DIGE analysis included alpha enolase, N(G),N(G)-dimethyl arginine dimethylaminohydrolase-1, s-arrestin, GAPDH, and carbonic anhydrase 2 (Table 3).

**Table 3:** **Differentially expressed proteins in the retina as identified by 2D DIGE.** Table shows the identification of protein spots highlighted in Fig. 2 following mass spectral analysis. Fold differences in expression between normal retina (N), and gliotic retina (G) are shown.

Spot	Uniprot ID	Accession	Peptide count	Protein name	Fold difference
					G/N
Spot 788	AMPL_HUMAN	P28838	5	Cytosol aminopeptidase	2.06
Spot 1567	GFAP_HUMAN	P14136	38	Glial fibrillary acidic protein	2.05
Spot 1568	ENOA_HUMAN	P06733	29	Alpha-enolase	0.45
Spot 891	ENOA_HUMAN	P06733	25	Alpha-enolase	0.43
Spot 1561	DDAH1_HUMAN	O94760	16	N(G),N(G)-dimethylarginine dimethylaminohydrolase 1	0.45
Spot 1603	CAH2_HUMAN	P00918	15	Carbonic anhydrase 2	0.43
Spot 820	ARRS_HUMAN	P10523	11	S-arrestin	0.27
Spot 1553	ARRS_HUMAN	P10523	3	S-arrestin	0.26
Spot 1080	DDAH1_HUMAN	O94760	4	N(G),N(G)-dimethylarginine dimethylaminohydrolase 1	0.49
Spot 831	ARRS_HUMAN	P10523	3	S-arrestin	0.38
Spot 1125	G3P_HUMAN	P04406	28	GAPDH	0.32
Spot 1005	VIME_HUMAN	P08670	44	Vimentin	2.67
Spot 759	PDIA1_HUMAN	P07237	17	Protein disulphide-isomerase	1.30
Spot 943	VIME_HUMAN	P08670	66	Vimentin	2.13

### 3.4 Gene ontology and pathway classification and enrichment analysis of differentially expressed proteins in the retina

**Fig. 4:**
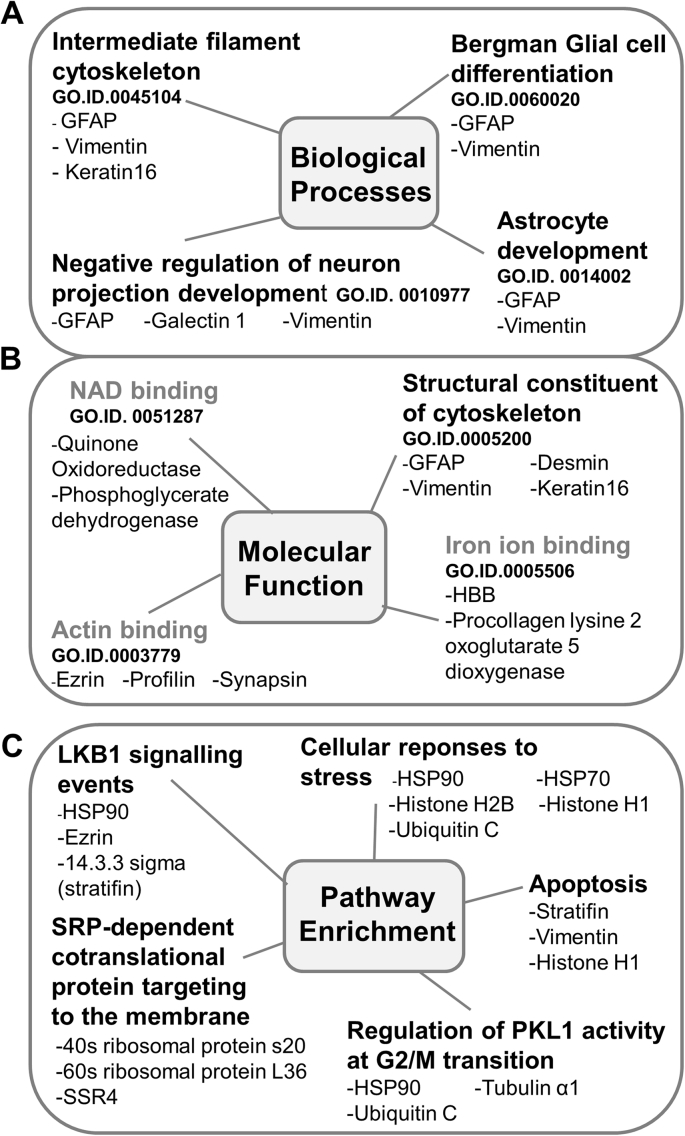
**Gene ontology and pathway analysis of proteins identified to be upregulated in the gliotic retina as compared to normal retina.** Figures have been simplified from WebGestalt GO, and IMPALA analysis results to show the classification and enrichment analysis of proteins found to be 2-fold upregulated in the gliotic retina as compared to normal retina. Gene ontology classifications highlighted in black represent statistically significant enriched gene ontology categories (P < 0.05) (Benjamin and Hochberg false discovery rate correction), whereas those highlighted grey indicate those which are of the top 10 non-significantly enriched categories where P > 0.05. Diagram shows GO categories and proteins assigned to those categories for (A) Biological processes (B) Molecular function. Similarly pathway analysis (C) shows the most significantly enriched signalling pathways of proteins 2-fold upregulated in the gliotic retina.

Proteins upregulated in gliotic retina compared with normal retina were subject to gene ontology (GO) enrichment analysis to identify key areas of interest. Proteins grouped according to the GO term ‘biological processes’ revealed an over-representation associated with the intermediate filament cytoskeleton, astrocyte development, glial cell differentiation and negative regulation of neuron projection development (Fig. 4A) and included GFAP, vimentin and galectin 1. Similarly, enrichment analysis of upregulated proteins classified according to molecular function, showed structural constituents of the cytoskeleton significantly enriched in the gliotic retina (Fig. 4B) and included GFAP, vimentin, keratin and desmin. Other protein groups classified by molecular function found enriched, although not statistically significant, included iron-ion binding, NAD binding and actin binding proteins (Fig. 4B). Proteins upregulated in the gliotic retina as compared to normal retina were subject to IMPaLA pathway analysis. (The top 12 significantly over-represented pathways are shown in Supplementary Table 3). The Liver Kinase B1- also known as Serine/Threonine Kinase 11 - STK11 (LKB1) signalling was the pathway most significantly represented within the group, and included the proteins HSP90aa1, ezrin, and 14.3.3δ. Other pathways highly represented included cellular responses to stress, regulation of serine/threonine-protein kinase 13 activity, also known as polo-like kinase 1 (PLK-1), G2/M transition and apoptosis (Fig. 4C).

**Fig. 5:**
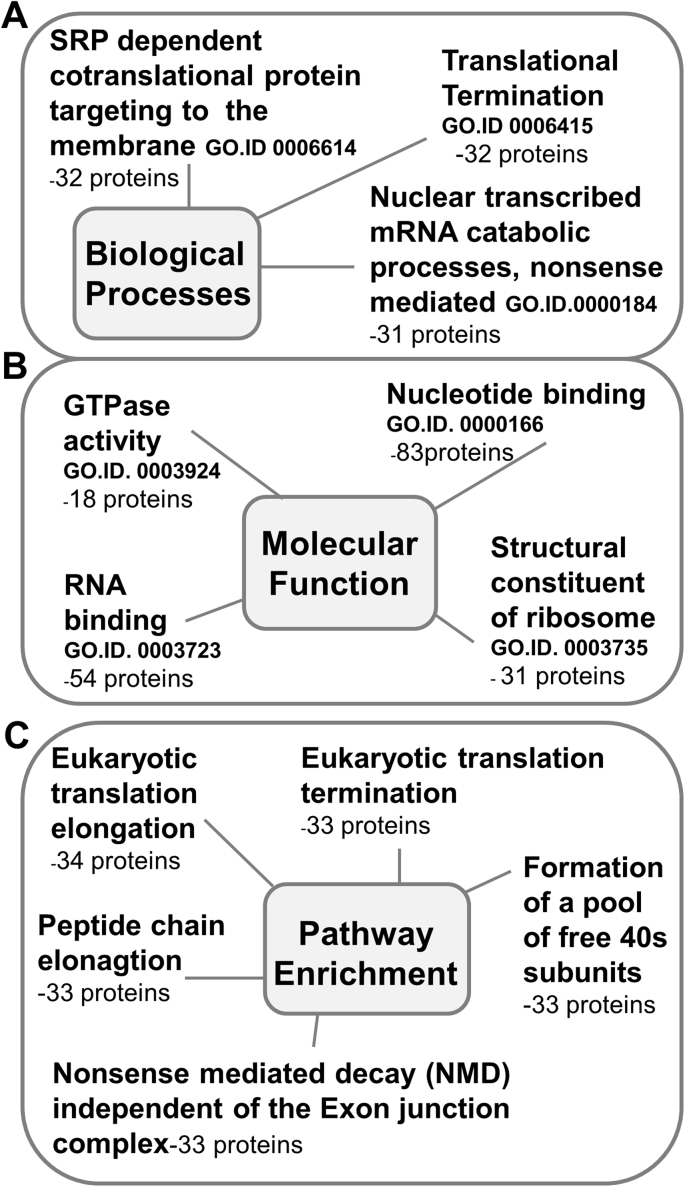
**Gene ontology analysis of proteins identified to be downregulated in the gliotic retina as compared to normal retina**. Figures have been simplified from WebGestalt GO results to show the classification and enrichment analysis of proteins found to be < 0.5-fold downregulated in the gliotic retina as compared to normal retina. Gene ontology classifications highlighted in black represent statistically significant enriched gene ontology categories (P < 0.05) (Benjamin and Hochberg false discovery rate correction). Diagram shows GO categories and proteins assigned to those categories for (A) Biological processes (B) Molecular function. Similarly, pathway analysis (C) shows the most significantly enriched pathways of the proteins which were 2-fold downregulated in the gliotic retina.

Of the proteins downregulated in the gliotic retina, classification according to biological processes revealed an over-representation of protein groups associated with bacterial signal recognition particle (SRP)-dependent co-translational protein targeting the membrane, mRNA catabolic processes, and translational termination (Fig. 5A). Enrichment analysis for downregulated proteins classified according to molecular function showed that GTPase activity, RNA binding, nucleotide binding, and structural constituents of the ribosome were also over-represented (Fig. 5B). Pathway analysis was also conducted for proteins shown to be downregulated in the gliotic retina as compared to normal retina. (The top 12 most significantly downregulated pathways are shown in Supplementary Table 4). Eukaryotic translation elongation was most significantly represented within the group and included mainly ribosomal related proteins. Other pathways highly represented included eukaryotic translation termination, peptide chain elongation and nonsense mediated decay independent of the exon junction complex (Fig. 5C).

### 3.5 Validation of retinal proteomic findings and localization of identified key proteins in Müller glia

**Fig. 6:**
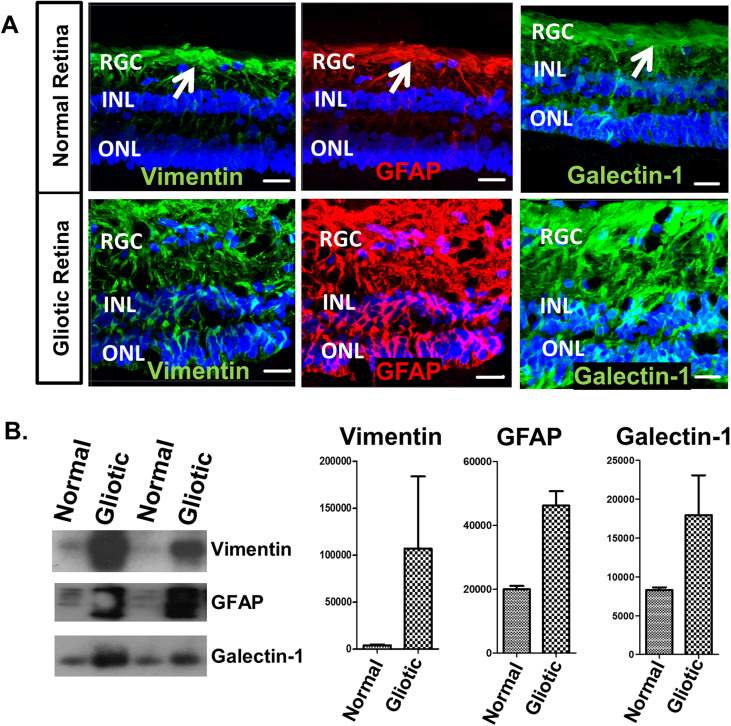
**Validation of proteomic profile (A)** Confocal images showing the expression of vimentin, GFAP and Galectin 1 by normal and gliotic human retina. As compared with normal retina, the gliotic retina showed a widespread and more intense staining for all three proteins, which was localized to Müller glia. White arrows show Müller end-feet; RGC = retinal ganglion cell layer; INL = inner nuclear layer; ONL = outer nuclear layer. Sections counterstained with DAPI to visualize retinal lamination. Scale bars: 20 μm **(B)** Western blot gels and corresponding histograms show an increase in vimentin, GFAP and Galectin-1 in the gliotic retinal specimens.

To validate our proteomics results we conducted western blot and immunofluorescence analyses. Using frozen sections of normal human retina (cadaveric) and gliotic specimens (PVR), we analysed the expression of the proteins GFAP, vimentin and galectin-1. Although the lamination of the gliotic retina examined was well preserved in the specimens examined, the overall morphological appearance is more irregular than that of the normal retina (Fig. 6A). Immunostaining of these specimens showed that Vimentin and GFAP was confined to the Müller glia end-feet region (nerve fibre layer; white arrows) in the normal retina. In comparison, Vimentin and GFAP immunostaining of PVR retina showed a widespread distribution of these proteins in Müller glia throughout the whole retinal section, this is one of the major characteristics of retinal gliosis (Fig. 6A). Immunostaining of normal retina for Galectin-1 was observed associated to Müller glia, more predominantly in the Müller end foot region, and spanning across all the layers of the retina. However, a more intense and widespread staining for this molecule was observed in Müller glia throughout the whole PVR retina (Fig. 6A). Similarly, western blot analysis of protein lysates from normal and gliotic retina showed an increased abundance of Vimentin, GFAP and Galectin-1 in the gliotic retina when compared with the normal retina (Fig. 6B).

## 4 Discussion

This study compared the protein expression profile of Müller glia with that of normal and gliotic PVR human retina and identified changes in cytoskeletal related proteins, extracellular matrix proteins and ribosomal proteins that occur during retinal gliosis. It would be important to analyse the proteome of Müller glia isolated from PVR gliotic retina with cells isolated from normal retina. However, in our laboratory, attempts to isolate Müller glia from PVR gliotic retina have been unsuccessful and we have been unable to obtain a stable culture of these cells despite many attempts (unpublished data). We hypothesise that this may be due to the gliotic nature of the tissue and the associated Müller glial changes that occur during gliosis (Bringmann et al., 2009). For the current study, we therefore sought samples of PVR gliotic retina to compare these with normal retina and established human Müller glial cell lines isolated from normal retina. The results showed important variations in key proteins expressed in Müller glia and normal and gliotic retina that highlight a potential significance of these molecules during retina degeneration.

Many cytoskeletal proteins, including ezrin and desmin, which are highly expressed by established human Müller glial cell lines, although not classified in the top 30 proteins, were found >2-fold upregulated in the gliotic retina. Upregulation of intermediate filament proteins, including GFAP and vimentin are generally accepted as indicators of retinal stress and Müller glial cell reactivity during retinal gliosis (Grosche et al., 1995; Lewis and Fisher, 2003), and are further supported by observations in vimentin and GFAP deficient mice, in which decreased photoreceptor degeneration and attenuated glial reactions are observed (Nakazawa et al., 2007). Ezrin, a peripheral membrane protein which aids in the organisation of cell morphology (Vaheri et al., 1997), has been found to be upregulated in a rabbit model of PVR (Zhou et al., 2012), suggesting that this protein may also be associated with human retinal gliosis. In zebrafish, Müller glia expression of GFAP is usually upregulated after retinal damage (Thomas et al., 2015), but upon regeneration GFAP expression is downregulated (Thummel et al., 2008). This suggests that GFAP may be more intricately regulated in Müller glia than first thought and that its expression may be potentially associated to the non-regenerative ability of these cells. These findings support previous observations of GFAP upregulation in the human gliotic retina, and highlight the importance of Müller cell structural changes in retinal remodelling during gliosis. These observations also indicate the importance to investigate the regulation of intermediate filament changes in human Müller glia that lead to these cells entering a gliotic state in favour of a progenitor-like state.

It is documented that extracellular matrix remodelling and gliosis occur in retinal diseases such as glaucoma (Wallace et al., 2014; Yan et al., 2000), diabetic retinopathy (Ioachim et al., 2005), PVR (Kon et al., 1998) and AMD (Nita et al., 2014). The findings that factors such as Tenascin C, PLOD2, CD166 and galectin1 are highly upregulated in gliotic retina, are therefore supported by these studies. Tenascin C is a glycoprotein involved in tissue remodelling and is known to support growth and differentiation. It is expressed in the ECM during development and injury (Jones and Jones, 2000) and it is found upregulated during diabetic retinopathy (Spirin et al., 1999). It has been postulated that Tenascin contributes to mechanical stiffness of the retina (Davis et al., 2012) and it is often co-expressed with matrix metalloproteinases (MMPs) (Jian et al., 2001), suggesting its importance in the deposition of the extracellular matrix during reactive gliosis. Upregulation of Tenascin C has also been linked to growth factors and cytokines such as TGFβ1, interleukin 1 and TNFα (Rettig et al., 1994), which have been associated with retinal degenerative disorders (Eastlake et al., 2016), suggesting that Tenascin C may contribute to gliosis. Galectin-1, a beta-galactoside-binding protein that plays an important role in cell adhesion and proliferation (Camby et al., 2006), has been previously implicated in retinal disease. Neutralising galectin-1 activity in rat eyes is reported to increase susceptibility to retinal detachment due to weak RPE and neural retinal adhesion (Uehara et al., 2001). Although upregulation of this protein could promote neural retina-RPE integrity, it may also induce retinal traction that perpetuates retinal detachment in PVR. Since Müller glia was observed to highly express Galectin 1 within the retina (Fig. 6), it is possible that these cells are the main source of this protein in the retina and studies are needed to determine the role of this protein during gliosis. Although the cell adhesion molecule CD166 (also known as ALCAM) is expressed by retinal ganglion cell axons during retinal development (Avci et al., 2004; Diekmann and Stuermer, 2009), its role during retinal degeneration has yet to be explored and constitutes a valid target for future investigations.

The observations that retinal specific proteins such as rhodopsin, s-arrestin, recoverin, retinaldehyde binding protein, Thy1 and rod cGMP, were severely downregulated in the gliotic retina, may reflect the neural cell death observed during gliosis. The findings that retinoschisin, an extracellular protein that plays a crucial role in the cellular organisation of the retina, and glutamine synthetase, an enzyme that plays a key metabolic role within the retina, were also markedly decreased in the gliotic retina may indicate key metabolic changes occurring in Müller glia during gliosis (Grosche et al., 1995; Lewis et al., 1989; Reid and Farber, 2005). Since downregulation of reticulon 4 (Neurite outgrowth inhibitor/Nogo) enhances neuronal cell growth and survival in the mouse retina (Kim et al., 2003), the present findings that Nogo was downregulated in the gliotic retina may indicate the existence of endogenous mechanisms triggered during gliosis to protect neurons from further damage. Although the present study does not provide evidence for the cells involved in the downregulation of Nogo, or the mechanisms underlying its decreased expression in the gliotic retina, it justifies further studies into the mechanisms that regulate the expression of these factors in the developed retina.

Pathway analysis studies showed that LKB1 signalling was positively regulated in the gliotic retina as compared to normal retina. LKB1 signalling is involved in the control of cell growth and metabolism by phosphorylation of the AMPK metabolic sensor (Lizcano et al., 2004), whilst HSP90AA1 bind and stabilise LKB1, possibly enhancing LKB1 signalling and cell growth inhibition (Gaude et al., 2012). Increased LKB1 signalling could lead to cell growth arrest and its upregulation during gliosis may reflect the damage observed. Several pathways involved in ribosomal processes were negatively regulated in the gliotic retina as compared to normal retina, suggesting that downregulation of the ribosomal subunits 40s and 60s may indicate a reduction in mRNA processing and protein translation during gliosis. In addition, loss of ribosome biogenesis is associated with ribosome stress, triggering activation of the p53 apoptotic pathway (Wang et al., 2015), therefore the loss of ribosome function could be ascribed to cell death occurring during gliosis and investigations into the activation of these pathways in Müller glia may provide important information on how these cells are affected during gliosis.

Müller glia markers identified in Müller cell lysates included GFAP, vimentin, glutamine synthetase, CD44 antigen and retinaldehyde binding protein 1 (CRALBP). It is reported that retinal Müller glia lose these *in vivo* features and undergo senescence with passaging in culture (Sarthy et al., 1998). Porcine Müller glia become fibroblastic after three days in culture and show upregulation of proteins associated with cytoskeleton, motility and proliferation, while downregulating those involved in transmitter recycling, glycolysis and detoxification (Hauck et al., 2003). In contrast, Müller glia that exhibit stem cell properties maintain their characteristic morphology (Limb et al., 2002) and although they may undergo adaptation changes to culture environments, the present results showed that immortalized Müller cell lines express specific markers associated with Müller glia at high levels. This correlates with previous observations that Müller stem cells continue to express Müller glia markers after 100 passages (Limb et al., 2002). We also identified proteins that have not previously reported to be expressed by Müller glia, including prelamin A/C, elongation factor 1, and serpin H1, for which it will be important to assess the functions of these factors during retinal gliosis. Additionally, markers highly expressed by Müller glia, including vimentin, GFAP, HSP90AA1 and poly-ubiquitin C, were also found >2-fold upregulated in the gliotic retina. Ubiquitination is one of the most important post-translational modifications of proteins and is involved in various cellular activities, such as protein degradation, apoptosis, DNA transcription and repair, cell division, neural degeneration and stress responses amongst others (Inobe and Nozaki, 2016). It is possible that the main source of poly-ubiquitin C found in the gliotic retina is Müller glia, thus highlighting the contribution of these cells to the gliotic changes that occur in the retina after disease or injury. Controlling ubiquitination may therefore constitute an approach to control gliosis and merits further investigations.

The issue of missing data values in label free proteomics analysis via mass spectrometry is widely discussed in the literature and considered a major concern (Lazar et al., 2016). Analysis of our dataset was processed through Progenesis software, which has been reported to generate consistent accurate results and very few missing data values (Valikangas et al., 2017). Although this software does not give a ‘missing data rate’ it does however produce some hits which are presented as having zero abundance which could be considered missing data and therefore these were excluded from our study.

In the present study it is possible that factors expressed by Müller glia may be under-represented in the retinal samples due to the presence of other retinal cell types. Nevertheless, variations in key proteins expressed in Müller glia and normal and gliotic retina highlight the importance of these molecules in retina degeneration. It also paves the way for further investigations into mechanisms that control the expression of gliosis associated proteins by Müller glia and their potential role in the prevention or promotion of endogenous regeneration of the adult mammalian retina.

## Funding sources

The study was supported by the MRC grant Refs. MR/K008722/1 and MR/P01660X/1; Moorfields Eye Charity and the NIHR Biomedical Research Centre at Moorfields Eye Hospital and UCL Institute of Ophthalmology, London, UK. WEH is funded by the Great Ormond street Biomedical Research Centre.
