# Supplementary material for: Comparative proteomic analysis of normal and gliotic PVR retina and contribution of Müller glia to this profile
Source: Exp Eye Res. 2018 Dec;177:197–207. doi: 10.1016/j.exer.2018.08.016 (PMC6280037; doi:10.1016/j.exer.2018.08.016)
Supplement: Multimedia component 1 [file mmc1.docx]

|  |  |  |  |  | Expression | | |
| --- | --- | --- | --- | --- | --- | --- | --- |
| Accession | Peptides (unique) | score | P-value | Description | gliotic | normal | muller |
| QOR_HUMAN | 4 | 12.68 | 5.97 | Quinone oxidoreductase OS Homo sapiens GN CRYZ PE 1 SV 1 | 16132.53 | 1750.47 | 1279.71 |
| CD166_HUMAN | 3 | 12.23 | 25.6 | CD166 antigen OS Homo sapiens GN ALCAM PE 1 SV 2 | 53.9316 | 6.58 | 168.43 |
| HBB_HUMAN | 77 | 486.75 | 5.17 | Hemoglobin subunit beta OS Homo sapiens GN HBB PE 1 SV 2 | 24265 | 2984.09 | 2225.01 |
| HBA_HUMAN | 50 | 272.67 | 3.47 | Hemoglobin subunit alpha OS Homo sapiens GN HBA1 PE 1 SV 2 | 12159.32 | 1662.77 | 1723.38 |
| CNPY2_HUMAN | 2 | 12.51 | 15.59 | Protein canopy homolog 2 OS Homo sapiens GN CNPY2 PE 1 SV 1 | 30.384 | 4.45 | 69.34 |
| HS71L_HUMAN | 32 (1) | 154.07 | 3.17 | Heat shock 70 kDa protein 1 like OS Homo sapiens GN HSPA1L PE 1 SV 2 | 51.2941 | 7.66 | 15.95 |
| SYN1_HUMAN | 2 | 6.12 | 2.55 | Synapsin 1 OS Homo sapiens GN SYN1 PE 1 SV 3 | 275.5449 | 51.13 | 57.22 |
| EZRI_HUMAN | 20 (2) | 84.18 | 8.96 | Ezrin OS Homo sapiens GN EZR PE 1 SV 4 | 93.0299 | 17.71 | 158.65 |
| DESM_HUMAN | 56 (2) | 192.75 | 2.5 | Desmin OS Homo sapiens GN DES PE 1 SV 3 | 810.9785 | 167.56 | 418.61 |
| GFAP_HUMAN | 217 (138) | 955.68 | 2.4 | Glial fibrillary acidic protein OS Homo sapiens GN GFAP PE 1 SV 1 | 40301 | 8965.61 | 7957.37 |
| 1433S_HUMAN | 21 (3) | 63.2 | 11.17 | 14 3 3 protein sigma OS Homo sapiens GN SFN PE 1 SV 1 | 105.9642 | 24.54 | 274.12 |
| RL36_HUMAN | 5 | 24.01 | 2.45 | 60S ribosomal protein L36 OS Homo sapiens GN RPL36 PE 1 SV 3 | 10251.6 | 2627.63 | 1981.62 |
| H2B1O_HUMAN | 143 (5) | 491.63 | 1.8 | Histone H2B type 1 O OS Homo sapiens GN HIST1H2BO PE 1 SV 3 | 437.7828 | 115.29 | 152.3 |
| K1C16_HUMAN | 18 (1) | 57.55 | 1.8 | Keratin type I cytoskeletal 16 OS Homo sapiens GN KRT16 PE 1 SV 4 | 118.8352 | 35.31 | 63.74 |
| SMD3_HUMAN | 4 (3) | 12.06 | 5.42 | Small nuclear ribonucleoprotein Sm D3 OS Homo sapiens GN SNRPD3 PE 1 SV 1 | 192.4953 | 68.09 | 368.74 |
| TBA1A_HUMAN | 199 (10) | 1038.83 | 1.46 | Tubulin alpha 1A chain OS Homo sapiens GN TUBA1A PE 1 SV 1 | 3387.584 | 1284.25 | 1096.49 |
| TENA_HUMAN | 2 | 11.69 | 23.14 | Tenascin OS Homo sapiens GN TNC PE 1 SV 3 | 38.4653 | 15.17 | 351 |
| VIME_HUMAN | 432 (297) | 2074.52 | 2.31 | Vimentin OS Homo sapiens GN VIM PE 1 SV 4 | 73006 | 28900 | 66700 |
| RS20_HUMAN | 2 | 20.73 | 13.82 | 40S ribosomal protein S20 OS Homo sapiens GN RPS20 PE 1 SV 1 | 28.485 | 12.28 | 169.76 |
| PLOD2_HUMAN | 6 | 30.77 | 55.68 | Procollagen lysine 2 oxoglutarate 5 dioxygenase 2 OS Homo sapiens GN PLOD2 PE 1 SV 2 | 17.2387 | 7.47 | 416.02 |
| SSRD_HUMAN | 4 (3) | 18.86 | 20.93 | Translocon associated protein subunit delta OS Homo sapiens GN SSR4 PE 1 SV 1 | 20.0239 | 9.24 | 193.28 |
| SERA_HUMAN | 3 (2) | 18.67 | 6.87 | D 3 phosphoglycerate dehydrogenase OS Homo sapiens GN PHGDH PE 1 SV 4 | 16.0993 | 7.57 | 52 |
| UBC_HUMAN | 37 (35) | 193.67 | 2.65 | Polyubiquitin C OS Homo sapiens GN UBC PE 1 SV 3 | 3173.166 | 1544.08 | 3987.6 |
| LEG1_HUMAN | 25 (23) | 135.32 | 6.03 | Galectin 1 OS Homo sapiens GN LGALS1 PE 1 SV 2 | 667.4352 | 329.36 | 1908.17 |
| PROF1_HUMAN | 21 | 135.35 | 17.93 | Profilin 1 OS Homo sapiens GN PFN1 PE 1 SV 2 | 405.5842 | 201.7 | 3446.2 |
| H13_HUMAN | 23 (2) | 102.86 | 1.05 | Histone H1 3 OS Homo sapiens GN HIST1H1D PE 1 SV 2 | 2420.719 | 1205.35 | 1188.72 |
| HS90A_HUMAN | 76 (36) | 384.36 | 1.37 | Heat shock protein HSP 90 alpha OS Homo sapiens GN HSP90AA1 PE 1 SV 5 | 6731.048 | 3370.26 | 4383.08 |
| LAMP2_HUMAN | 3 | 17.86 | 11.63 | Lysosome associated membrane glycoprotein 2 OS Homo sapiens GN LAMP2 PE 1 SV 2 | 50.6189 | 26.21 | 278.91 |
| SYG_HUMAN | 3 | 21.07 | 3.36 | Glycine tRNA ligase OS Homo sapiens GN GARS PE 1 SV 3 | 199.5005 | 107.37 | 317.89 |
| CD44_HUMAN | 5 | 19.9 | 3.12 | CD44 antigen OS Homo sapiens GN CD44 PE 1 SV 3 | 245.4352 | 132.32 | 363.46 |
| CRYAA_HUMAN | 37 (32) | 219.16 | 4.25 | Alpha crystallin A chain OS Homo sapiens GN CRYAA PE 1 SV 2 | 6943.672 | 3786.38 | 890.37 |
| CALM_HUMAN | 5 | 44.62 | 3.37 | Calmodulin OS Homo sapiens GN CALM1 PE 1 SV 2 | 234.6531 | 133.33 | 374.78 |
| RL35_HUMAN | 2 | 5.55 | 6.21 | 60S ribosomal protein L35 OS Homo sapiens GN RPL35 PE 1 SV 2 | 48.9098 | 28.87 | 144.01 |
| CO1A1_HUMAN | 32 (31) | 141.98 | 15.18 | Collagen alpha 1 I chain OS Homo sapiens GN COL1A1 PE 1 SV 5 | 410.8803 | 243.21 | 2955.9 |
| RAB35_HUMAN | 3 (2) | 12.34 | 6.51 | Ras related protein Rab 35 OS Homo sapiens GN RAB35 PE 1 SV 1 | 79.4204 | 48.04 | 245.23 |
| CRYAB_HUMAN | 22 | 73.48 | 3.85 | Alpha crystallin B chain OS Homo sapiens GN CRYAB PE 1 SV 2 | 1614.783 | 999.66 | 2943.51 |
| H4_HUMAN | 158 (154) | 640.41 | 2.38 | Histone H4 OS Homo sapiens GN HIST1H4A PE 1 SV 2 | 30384 | 18900 | 34300 |
| SERPH_HUMAN | 54 (51) | 350.27 | 10.83 | Serpin H1 OS Homo sapiens GN SERPINH1 PE 1 SV 2 | 1531.987 | 964.89 | 7862.46 |
| NPM_HUMAN | 11 | 37.51 | 2.05 | Nucleophosmin OS Homo sapiens GN NPM1 PE 1 SV 2 | 913.0392 | 579.08 | 885.83 |
| PDIA4_HUMAN | 34 (30) | 183.25 | 5.77 | Protein disulfide isomerase A4 OS Homo sapiens GN PDIA4 PE 1 SV 2 | 1074.201 | 682.67 | 2938.14 |
| TM109_HUMAN | 2 | 11.83 | 9.4 | Transmembrane protein 109 OS Homo sapiens GN TMEM109 PE 1 SV 1 | 28.9281 | 18.4 | 128.85 |
| CO6A1_HUMAN | 9 | 57.99 | 9.51 | Collagen alpha 1 VI chain OS Homo sapiens GN COL6A1 PE 1 SV 3 | 139.7242 | 89.56 | 629.47 |
| CH10_HUMAN | 12 | 80.36 | 9.67 | 10 kDa heat shock protein mitochondrial OS Homo sapiens GN HSPE1 PE 1 SV 2 | 190.744 | 123.41 | 873.89 |
| CATB_HUMAN | 2 | 5.49 | 1.4 | Cathepsin B OS Homo sapiens GN CTSB PE 1 SV 3 | 547.3129 | 363.18 | 347.17 |
| S10A6_HUMAN | 21 | 88.04 | 14.84 | Protein S100 A6 OS Homo sapiens GN S100A6 PE 1 SV 1 | 418.0121 | 278.47 | 2939.48 |
| K2C1_HUMAN | 144 (112) | 773.53 | 1.41 | Keratin type II cytoskeletal 1 OS Homo sapiens GN KRT1 PE 1 SV 6 | 14627.49 | 9769.06 | 8529.87 |
| MOES_HUMAN | 28 (9) | 126.52 | 3.64 | Moesin OS Homo sapiens GN MSN PE 1 SV 3 | 324.1382 | 216.93 | 558.98 |
| CO1A2_HUMAN | 31 | 150.51 | 6.61 | Collagen alpha 2 I chain OS Homo sapiens GN COL1A2 PE 1 SV 7 | 592.7412 | 401.28 | 1856.14 |
| ALBU_HUMAN | 2 | 11.06 | 2.34 | Serum albumin OS Homo sapiens GN ALB PE 1 SV 2 | 151.6246 | 103.67 | 44.24 |
| ACTBM_HUMAN | 32 (1) | 165.35 | 9.67 | Putative beta actin like protein 3 OS Homo sapiens GN POTEKP PE 5 SV 1 | 87.5017 | 60.65 | 400.99 |
| PLOD1_HUMAN | 17 | 95.8 | 22.14 | Procollagen lysine 2 oxoglutarate 5 dioxygenase 1 OS Homo sapiens GN PLOD1 PE 1 SV 2 | 80.0534 | 57.52 | 840.17 |
| RABP1_HUMAN | 38 (32) | 184.92 | 8.65 | Cellular retinoic acid binding protein 1 OS Homo sapiens GN CRABP1 PE 1 SV 2 | 2530.692 | 1830.24 | 211.53 |
| RS18_HUMAN | 4 | 23.56 | 9.3 | 40S ribosomal protein S18 OS Homo sapiens GN RPS18 PE 1 SV 3 | 66.4439 | 48.73 | 292.96 |
| EAA1_HUMAN | 13 | 77.06 | 13.75 | Excitatory amino acid transporter 1 OS Homo sapiens GN SLC1A3 PE 1 SV 1 | 1828.948 | 1345.91 | 97.86 |
| CKAP4_HUMAN | 33 (32) | 197.74 | 7.23 | Cytoskeleton associated protein 4 OS Homo sapiens GN CKAP4 PE 1 SV 2 | 847.2072 | 623.63 | 2904.6 |
| PDIA6_HUMAN | 12 | 75.8 | 4.56 | Protein disulfide isomerase A6 OS Homo sapiens GN PDIA6 PE 1 SV 1 | 696.7853 | 515.69 | 1506.15 |
| TBA1B_HUMAN | 213 (4) | 1090.15 | 1.56 | Tubulin alpha 1B chain OS Homo sapiens GN TUBA1B PE 1 SV 1 | 254.677 | 188.8 | 123.38 |
| NDKB_HUMAN | 25 (6) | 118.88 | 18.94 | Nucleoside diphosphate kinase B OS Homo sapiens GN NME2 PE 1 SV 1 | 51.6528 | 39.39 | 463.8 |
| LRC59_HUMAN | 2 | 14.64 | 11.48 | Leucine rich repeat containing protein 59 OS Homo sapiens GN LRRC59 PE 1 SV 1 | 65.3256 | 49.96 | 355.46 |
| LMNA_HUMAN | 84 (78) | 553.77 | 5.94 | Prelamin A C OS Homo sapiens GN LMNA PE 1 SV 1 | 5016.609 | 3886.55 | 14100 |
| CLIC1_HUMAN | 5 | 18.17 | 8.07 | Chloride intracellular channel protein 1 OS Homo sapiens GN CLIC1 PE 1 SV 4 | 44.0779 | 34.74 | 168.62 |
| HS90B_HUMAN | 82 (29) | 493.58 | 4.48 | Heat shock protein HSP 90 beta OS Homo sapiens GN HSP90AB1 PE 1 SV 4 | 1510.043 | 1192.78 | 3205.21 |
| 1433T_HUMAN | 29 (9) | 126.53 | 2.72 | 14 3 3 protein theta OS Homo sapiens GN YWHAQ PE 1 SV 1 | 347.8546 | 279.42 | 448.89 |
| H2A2B_HUMAN | 79 (3) | 277.25 | 6.36 | Histone H2A type 2 B OS Homo sapiens GN HIST2H2AB PE 1 SV 3 | 110.7539 | 89.76 | 333.61 |
| CALR_HUMAN | 16 (15) | 77.48 | 4.59 | Calreticulin OS Homo sapiens GN CALR PE 1 SV 1 | 893.3951 | 738.93 | 1941.85 |
| RS5_HUMAN | 4 | 26.26 | 6.45 | 40S ribosomal protein S5 OS Homo sapiens GN RPS5 PE 1 SV 4 | 129.132 | 107.22 | 394.65 |
| RL18_HUMAN | 15 (14) | 70.49 | 3.66 | 60S ribosomal protein L18 OS Homo sapiens GN RPL18 PE 1 SV 2 | 743.9649 | 626.51 | 1289.46 |
| MYH9_HUMAN | 83 (80) | 461.07 | 8.14 | Myosin 9 OS Homo sapiens GN MYH9 PE 1 SV 4 | 1317.041 | 1119.49 | 5083.56 |
| PPIA_HUMAN | 43 (35) | 250.62 | 7.21 | Peptidyl prolyl cis trans isomerase A OS Homo sapiens GN PPIA PE 1 SV 2 | 1846.44 | 1583.28 | 6308.16 |
| BGH3_HUMAN | 6 | 39.79 | 10.02 | Transforming growth factor beta induced protein ig h3 OS Homo sapiens GN TGFBI PE 1 SV 1 | 53.0243 | 45.47 | 251.8 |
| PAL4A_HUMAN | 6 (2) | 27.21 | 3.09 | Peptidyl prolyl cis trans isomerase A like 4A B C OS Homo sapiens GN PPIAL4A PE 1 SV 1 | 49.7327 | 42.9 | 72.95 |
| PDIA1_HUMAN | 39 (37) | 202.22 | 6.25 | Protein disulfide isomerase OS Homo sapiens GN P4HB PE 1 SV 3 | 1341.559 | 1158.38 | 3972.13 |
| TBB6_HUMAN | 72 (3) | 356.18 | 1.83 | Tubulin beta 6 chain OS Homo sapiens GN TUBB6 PE 1 SV 1 | 168.9266 | 146.24 | 97.58 |
| HSP76_HUMAN | 20 (1) | 101.34 | 2.46 | Heat shock 70 kDa protein 6 OS Homo sapiens GN HSPA6 PE 1 SV 2 | 94.0005 | 81.41 | 109.76 |
| GRP78_HUMAN | 70 (57) | 432.13 | 7.67 | 78 kDa glucose regulated protein OS Homo sapiens GN HSPA5 PE 1 SV 2 | 2852.868 | 2485.39 | 10400 |
| TAGL2_HUMAN | 15 | 91.16 | 18.43 | Transgelin 2 OS Homo sapiens GN TAGLN2 PE 1 SV 3 | 194.3943 | 170.94 | 1697.99 |
| SF3B3_HUMAN | 2 | 5.71 | 10.85 | Splicing factor 3B subunit 3 OS Homo sapiens GN SF3B3 PE 1 SV 4 | 113.3492 | 100.89 | 582.78 |
| HNRPQ_HUMAN | 5 (1) | 31.69 | 6.06 | Heterogeneous nuclear ribonucleoprotein Q OS Homo sapiens GN SYNCRIP PE 1 SV 2 | 26.6915 | 23.87 | 76.73 |
| HYOU1_HUMAN | 26 | 143.22 | 6.61 | Hypoxia up regulated protein 1 OS Homo sapiens GN HYOU1 PE 1 SV 1 | 471.7749 | 425.44 | 1477.37 |
| K1C9_HUMAN | 29 (21) | 136.82 | 1.91 | Keratin type I cytoskeletal 9 OS Homo sapiens GN KRT9 PE 1 SV 3 | 1652.974 | 1496.9 | 914.2 |
| GLU2B_HUMAN | 11 | 57.47 | 7.89 | Glucosidase 2 subunit beta OS Homo sapiens GN PRKCSH PE 1 SV 2 | 155.2538 | 141.29 | 580.74 |
| PRDX5_HUMAN | 3 | 17.89 | 4.56 | Peroxiredoxin 5 mitochondrial OS Homo sapiens GN PRDX5 PE 1 SV 4 | 68.8704 | 63.11 | 148.97 |
| TBB2A_HUMAN | 153 (5) | 824.59 | 1.94 | Tubulin beta 2A chain OS Homo sapiens GN TUBB2A PE 1 SV 1 | 439.7662 | 404.75 | 260.32 |
| ENOA_HUMAN | 226 (144) | 1132.28 | 1.97 | Alpha enolase OS Homo sapiens GN ENO1 PE 1 SV 2 | 30173 | 27800 | 14100 |
| RAB1A_HUMAN | 9 (3) | 51.39 | 2.43 | Ras related protein Rab 1A OS Homo sapiens GN RAB1A PE 1 SV 3 | 84.8853 | 79.34 | 97.59 |
| THIO_HUMAN | 2 | 11.98 | 11.15 | Thioredoxin OS Homo sapiens GN TXN PE 1 SV 3 | 28.8015 | 27.43 | 152.28 |
| ALDOC_HUMAN | 41 (32) | 285.85 | 4.64 | Fructose bisphosphate aldolase C OS Homo sapiens GN ALDOC PE 1 SV 2 | 4407.938 | 4250.06 | 916.46 |
| H31_HUMAN | 121 (119) | 264.1 | 2.64 | Histone H3 1 OS Homo sapiens GN HIST1H3A PE 1 SV 2 | 13058.26 | 12600 | 16400 |
| RPN1_HUMAN | 18 | 104.52 | 9.15 | Dolichyl diphosphooligosaccharide protein glycosyltransferase subunit 1 OS Homo sapiens GN RPN1 PE | 216.0218 | 212.43 | 936.79 |
| EF2_HUMAN | 29 | 146.08 | 5.66 | Elongation factor 2 OS Homo sapiens GN EEF2 PE 1 SV 4 | 962.3288 | 952.8 | 2582.59 |
| P3H1_HUMAN | 5 | 30.25 | 11.32 | Prolyl 3 hydroxylase 1 OS Homo sapiens GN LEPRE1 PE 1 SV 2 | 61.4221 | 60.84 | 329.54 |
| IF4A2_HUMAN | 4 (1) | 18.68 | 5.34 | Eukaryotic initiation factor 4A II OS Homo sapiens GN EIF4A2 PE 1 SV 2 | 27.0291 | 26.78 | 68.45 |
| ACTB_HUMAN | 191 (60) | 1004.41 | 3.96 | Actin cytoplasmic 1 OS Homo sapiens GN ACTB PE 1 SV 1 | 10585.85 | 10600 | 19800 |
| FKBP9_HUMAN | 4 | 12.08 | 3.61 | Peptidyl prolyl cis trans isomerase FKBP9 OS Homo sapiens GN FKBP9 PE 1 SV 2 | 244.8233 | 247.33 | 419.4 |
| ENPL_HUMAN | 82 (60) | 461.5 | 7.01 | Endoplasmin OS Homo sapiens GN HSP90B1 PE 1 SV 1 | 2347.881 | 2373.28 | 7801.21 |
| PDIA3_HUMAN | 33 (32) | 206.11 | 5.73 | Protein disulfide isomerase A3 OS Homo sapiens GN PDIA3 PE 1 SV 4 | 1545.09 | 1566.19 | 4194.06 |
| ENPLL_HUMAN | 11 (2) | 54.71 | 28.2 | Putative endoplasmin like protein OS Homo sapiens GN HSP90B2P PE 5 SV 1 | 10.7188 | 11.02 | 143.29 |
| PPIB_HUMAN | 43 (40) | 293.15 | 18.81 | Peptidyl prolyl cis trans isomerase B OS Homo sapiens GN PPIB PE 1 SV 2 | 974.5879 | 1023.13 | 8690.1 |
| GRP75_HUMAN | 26 | 151.97 | 2.81 | Stress 70 protein mitochondrial OS Homo sapiens GN HSPA9 PE 1 SV 2 | 1136.889 | 1194.19 | 1511.52 |
| TPIS_HUMAN | 96 (93) | 502.45 | 2.26 | Triosephosphate isomerase OS Homo sapiens GN TPI1 PE 1 SV 3 | 6900.185 | 7387.65 | 6457.41 |
| RL6_HUMAN | 4 | 28.22 | 3.22 | 60S ribosomal protein L6 OS Homo sapiens GN RPL6 PE 1 SV 3 | 264.2775 | 283.03 | 402.79 |
| HSP7C_HUMAN | 87 (41) | 539.37 | 3.8 | Heat shock cognate 71 kDa protein OS Homo sapiens GN HSPA8 PE 1 SV 1 | 2374.51 | 2574.73 | 4274.02 |
| ANXA6_HUMAN | 29 | 144.52 | 2.3 | Annexin A6 OS Homo sapiens GN ANXA6 PE 1 SV 3 | 1411.991 | 1537.02 | 928.02 |
| EF1G_HUMAN | 2 | 12.41 | 7.19 | Elongation factor 1 gamma OS Homo sapiens GN EEF1G PE 1 SV 3 | 68.5539 | 74.7 | 233.6 |
| RAP1A_HUMAN | 6 | 23.35 | 3.89 | Ras related protein Rap 1A OS Homo sapiens GN RAP1A PE 1 SV 1 | 184.7727 | 202.35 | 340.71 |
| EF1A1_HUMAN | 50 (46) | 293.38 | 5.27 | Elongation factor 1 alpha 1 OS Homo sapiens GN EEF1A1 PE 1 SV 1 | 3442.17 | 3773.34 | 8589.95 |
| CH60_HUMAN | 24 | 135.78 | 3.52 | 60 kDa heat shock protein mitochondrial OS Homo sapiens GN HSPD1 PE 1 SV 2 | 1088.57 | 1203.11 | 1818.42 |
| SYT1_HUMAN | 6 | 32.59 | 5.44 | Synaptotagmin 1 OS Homo sapiens GN SYT1 PE 1 SV 1 | 382.8173 | 423.23 | 77.75 |
| KPYM_HUMAN | 148 (141) | 800.09 | 2.35 | Pyruvate kinase isozymes M1 M2 OS Homo sapiens GN PKM PE 1 SV 4 | 18905.37 | 21100 | 15100 |
| RL8_HUMAN | 5 (3) | 20.11 | 14.77 | 60S ribosomal protein L8 OS Homo sapiens GN RPL8 PE 1 SV 2 | 22.4293 | 25.18 | 156.92 |
| ECHA_HUMAN | 2 (1) | 12.24 | 2.55 | Trifunctional enzyme subunit alpha mitochondrial OS Homo sapiens GN HADHA PE 1 SV 2 | 50.8088 | 57.26 | 61.39 |
| CALX_HUMAN | 29 (24) | 117.6 | 2.88 | Calnexin OS Homo sapiens GN CANX PE 1 SV 2 | 1406.547 | 1606.58 | 1922.41 |
| LMNB1_HUMAN | 13 (7) | 70.1 | 4 | Lamin B1 OS Homo sapiens GN LMNB1 PE 1 SV 2 | 183.9498 | 211.9 | 349 |
| TBB2B_HUMAN | 150 (3) | 825.68 | 2.44 | Tubulin beta 2B chain OS Homo sapiens GN TUBB2B PE 1 SV 1 | 246.6168 | 285.03 | 174.2 |
| ENOB_HUMAN | 61 (3) | 293.44 | 2.44 | Beta enolase OS Homo sapiens GN ENO3 PE 1 SV 5 | 276.6421 | 319.93 | 197.41 |
| H2B1D_HUMAN | 148 (34) | 468.67 | 3.94 | Histone H2B type 1 D OS Homo sapiens GN HIST1H2BD PE 1 SV 2 | 2034.842 | 2358.01 | 3802.9 |
| PRDX1_HUMAN | 52 (38) | 199.93 | 3.43 | Peroxiredoxin 1 OS Homo sapiens GN PRDX1 PE 1 SV 1 | 2058.242 | 2418.29 | 3342.28 |
| MYL6_HUMAN | 10 | 72.78 | 7.14 | Myosin light polypeptide 6 OS Homo sapiens GN MYL6 PE 1 SV 2 | 343.8245 | 405.99 | 1163.19 |
| RAB10_HUMAN | 4 (2) | 24.48 | 5.6 | Ras related protein Rab 10 OS Homo sapiens GN RAB10 PE 1 SV 1 | 27.3667 | 32.49 | 72.61 |
| RL18A_HUMAN | 2 | 12.38 | 12.78 | 60S ribosomal protein L18a OS Homo sapiens GN RPL18A PE 1 SV 2 | 15.2342 | 18.23 | 92.25 |
| GTR1_HUMAN | 9 | 54.8 | 11 | Solute carrier family 2 facilitated glucose transporter member 1 OS Homo sapiens GN SLC2A1 PE 1 SV | 1153.664 | 1388 | 126.23 |
| G3P_HUMAN | 125 (118) | 757.84 | 2.55 | Glyceraldehyde 3 phosphate dehydrogenase OS Homo sapiens GN GAPDH PE 1 SV 3 | 26797 | 32400 | 16100 |
| ANXA5_HUMAN | 60 (59) | 298.66 | 2.56 | Annexin A5 OS Homo sapiens GN ANXA5 PE 1 SV 2 | 4332.991 | 5260.05 | 4218.54 |
| PRDX2_HUMAN | 37 (30) | 141.87 | 2.58 | Peroxiredoxin 2 OS Homo sapiens GN PRDX2 PE 1 SV 5 | 1855.386 | 2270.61 | 1038.05 |
| TKT_HUMAN | 23 | 146.48 | 2.62 | Transketolase OS Homo sapiens GN TKT PE 1 SV 3 | 1081.312 | 1340.29 | 1089.44 |
| TRFE_HUMAN | 6 | 36.4 | 5.62 | Serotransferrin OS Homo sapiens GN TF PE 1 SV 3 | 318.7155 | 397.93 | 70.75 |
| PGAM1_HUMAN | 22 | 91.21 | 2.66 | Phosphoglycerate mutase 1 OS Homo sapiens GN PGAM1 PE 1 SV 2 | 1377.64 | 1734.97 | 1097.99 |
| H2AV_HUMAN | 58 (6) | 156.36 | 6.77 | Histone H2A V OS Homo sapiens GN H2AFV PE 1 SV 3 | 130.8411 | 164.91 | 419.68 |
| RL30_HUMAN | 5 | 26.59 | 23.2 | 60S ribosomal protein L30 OS Homo sapiens GN RPL30 PE 1 SV 2 | 34.7517 | 44.1 | 382.07 |
| QCR1_HUMAN | 3 | 19.36 | 3.05 | Cytochrome b c1 complex subunit 1 mitochondrial OS Homo sapiens GN UQCRC1 PE 1 SV 3 | 95.4353 | 123 | 137.88 |
| PGM2_HUMAN | 4 | 11.31 | 2.82 | Phosphoglucomutase 2 OS Homo sapiens GN PGM2 PE 1 SV 4 | 152.4053 | 197.4 | 70.11 |
| TCPQ_HUMAN | 4 | 24.31 | 4.9 | T complex protein 1 subunit theta OS Homo sapiens GN CCT8 PE 1 SV 4 | 143.0158 | 187.24 | 331.84 |
| RPN2_HUMAN | 4 | 27.58 | 4.71 | Dolichyl diphosphooligosaccharide protein glycosyltransferase subunit 2 OS Homo sapiens GN RPN2 PE | 139.9563 | 184.63 | 312.38 |
| ML12A_HUMAN | 6 (5) | 31.89 | 18.88 | Myosin regulatory light chain 12A OS Homo sapiens GN MYL12A PE 1 SV 2 | 54.0793 | 71.35 | 484.08 |
| K2C5_HUMAN | 4 (1) | 36.35 | 2.79 | Keratin type II cytoskeletal 5 OS Homo sapiens GN KRT5 PE 1 SV 3 | 44.3944 | 58.75 | 34.13 |
| CALU_HUMAN | 9 (8) | 64.36 | 18.36 | Calumenin OS Homo sapiens GN CALU PE 1 SV 2 | 128.4568 | 171.13 | 1118.07 |
| 1433E_HUMAN | 30 (17) | 136.36 | 2.82 | 14 3 3 protein epsilon OS Homo sapiens GN YWHAE PE 1 SV 1 | 897.9527 | 1199.17 | 1078.75 |
| IF4A1_HUMAN | 5 (2) | 25.65 | 7.45 | Eukaryotic initiation factor 4A I OS Homo sapiens GN EIF4A1 PE 1 SV 1 | 46.9053 | 63.64 | 165.51 |
| XRCC6_HUMAN | 13 (12) | 76.03 | 2.86 | X ray repair cross complementing protein 6 OS Homo sapiens GN XRCC6 PE 1 SV 2 | 351.3994 | 477.06 | 458.32 |
| GELS_HUMAN | 3 | 12.16 | 3.09 | Gelsolin OS Homo sapiens GN GSN PE 1 SV 1 | 171.4375 | 233.56 | 251.15 |
| TBA4A_HUMAN | 130 (14) | 731.78 | 3.06 | Tubulin alpha 4A chain OS Homo sapiens GN TUBA4A PE 1 SV 1 | 688.7673 | 939.22 | 307.43 |
| ALDOA_HUMAN | 50 (38) | 272.6 | 2.89 | Fructose bisphosphate aldolase A OS Homo sapiens GN ALDOA PE 1 SV 2 | 2916.505 | 3991.56 | 3722.94 |
| ANXA2_HUMAN | 63 (62) | 493.36 | 20.11 | Annexin A2 OS Homo sapiens GN ANXA2 PE 1 SV 2 | 1758.073 | 2412.2 | 16800 |
| PEBP1_HUMAN | 24 (23) | 107.63 | 2.9 | Phosphatidylethanolamine binding protein 1 OS Homo sapiens GN PEBP1 PE 1 SV 3 | 2188.64 | 3003.36 | 1261.94 |
| CATD_HUMAN | 26 (24) | 146.3 | 9.48 | Cathepsin D OS Homo sapiens GN CTSD PE 1 SV 1 | 459.6846 | 632.94 | 2064.55 |
| GBB2_HUMAN | 18 (1) | 59.48 | 3.83 | Guanine nucleotide binding protein G I G S G T subunit beta 2 OS Homo sapiens GN GNB2 PE 1 SV 3 | 10.0436 | 13.91 | 18.25 |
| RL12_HUMAN | 5 (4) | 25.89 | 17.9 | 60S ribosomal protein L12 OS Homo sapiens GN RPL12 PE 1 SV 1 | 23.9907 | 33.3 | 203.51 |
| G6PI_HUMAN | 18 | 110.25 | 2.97 | Glucose 6 phosphate isomerase OS Homo sapiens GN GPI PE 1 SV 4 | 1341.222 | 1890.59 | 844.99 |
| PYGB_HUMAN | 18 (11) | 77.31 | 2.98 | Glycogen phosphorylase brain form OS Homo sapiens GN PYGB PE 1 SV 5 | 459.2415 | 647.63 | 589.63 |
| SODC_HUMAN | 10 | 44.1 | 2.98 | Superoxide dismutase Cu Zn OS Homo sapiens GN SOD1 PE 1 SV 2 | 408.6437 | 577.34 | 380.19 |
| HSP71_HUMAN | 40 (9) | 193.36 | 3 | Heat shock 70 kDa protein 1A 1B OS Homo sapiens GN HSPA1A PE 1 SV 5 | 341.8833 | 485.79 | 331.71 |
| BASP1_HUMAN | 20 | 95.72 | 6.18 | Brain acid soluble protein 1 OS Homo sapiens GN BASP1 PE 1 SV 2 | 1127.901 | 1610.43 | 260.69 |
| CMC1_HUMAN | 4 | 16.91 | 3.07 | Calcium binding mitochondrial carrier protein Aralar1 OS Homo sapiens GN SLC25A12 PE 1 SV 2 | 132.7823 | 192.99 | 155.03 |
| TBB5_HUMAN | 161 (13) | 828.71 | 4.19 | Tubulin beta chain OS Homo sapiens GN TUBB PE 1 SV 2 | 1192.256 | 1736.95 | 2366.62 |
| TBB4B_HUMAN | 166 (6) | 888.55 | 6.13 | Tubulin beta 4B chain OS Homo sapiens GN TUBB4B PE 1 SV 1 | 957.4125 | 1396.49 | 227.95 |
| RB11B_HUMAN | 4 | 23.14 | 7.84 | Ras related protein Rab 11B OS Homo sapiens GN RAB11B PE 1 SV 4 | 64.6504 | 95.82 | 240.12 |
| AINX_HUMAN | 25 (6) | 65.34 | 3.19 | Alpha internexin OS Homo sapiens GN INA PE 1 SV 2 | 236.5521 | 357.24 | 273.43 |
| RLA0_HUMAN | 11 | 58.97 | 8.21 | 60S acidic ribosomal protein P0 OS Homo sapiens GN RPLP0 PE 1 SV 1 | 336.8826 | 517.4 | 1311.12 |
| ACTBL_HUMAN | 46 (3) | 215.25 | 3.41 | Beta actin like protein 2 OS Homo sapiens GN ACTBL2 PE 1 SV 2 | 178.0207 | 274.06 | 287.95 |
| K22E_HUMAN | 30 (10) | 176.58 | 3.25 | Keratin type II cytoskeletal 2 epidermal OS Homo sapiens GN KRT2 PE 1 SV 2 | 477.7673 | 736.85 | 295.69 |
| LDHA_HUMAN | 69 (58) | 321.58 | 3.9 | L lactate dehydrogenase A chain OS Homo sapiens GN LDHA PE 1 SV 2 | 3143.52 | 4863.98 | 5803.35 |
| H2A1D_HUMAN | 93 (3) | 324.73 | 7.64 | Histone H2A type 1 D OS Homo sapiens GN HIST1H2AD PE 1 SV 2 | 107.4412 | 167.51 | 388.79 |
| RHOC_HUMAN | 5 | 24.41 | 3.92 | Rho related GTP binding protein RhoC OS Homo sapiens GN RHOC PE 1 SV 1 | 128.3513 | 200.26 | 238.32 |
| RL11_HUMAN | 6 | 29.73 | 12.89 | 60S ribosomal protein L11 OS Homo sapiens GN RPL11 PE 1 SV 2 | 89.1053 | 140.29 | 544.33 |
| PGK1_HUMAN | 65 (61) | 404.49 | 3.34 | Phosphoglycerate kinase 1 OS Homo sapiens GN PGK1 PE 1 SV 3 | 5799.356 | 9182.77 | 3774.9 |
| SEPT7_HUMAN | 5 | 33.55 | 3.35 | Septin 7 OS Homo sapiens GN SEPT7 PE 1 SV 2 | 201.716 | 319.89 | 174.16 |
| SND1_HUMAN | 12 | 68.38 | 13.59 | Staphylococcal nuclease domain containing protein 1 OS Homo sapiens GN SND1 PE 1 SV 1 | 131.4108 | 211.56 | 846.03 |
| COF1_HUMAN | 23 (22) | 100.25 | 9.21 | Cofilin 1 OS Homo sapiens GN CFL1 PE 1 SV 3 | 712.3993 | 1149.41 | 3110.24 |
| TCPG_HUMAN | 3 | 19.18 | 3.74 | T complex protein 1 subunit gamma OS Homo sapiens GN CCT3 PE 1 SV 4 | 70.1997 | 113.42 | 124.43 |
| CAH2_HUMAN | 63 (60) | 348.86 | 8.59 | Carbonic anhydrase 2 OS Homo sapiens GN CA2 PE 1 SV 2 | 6824.774 | 11100 | 1290.05 |
| DPYL3_HUMAN | 11 (9) | 75.93 | 3.5 | Dihydropyrimidinase related protein 3 OS Homo sapiens GN DPYSL3 PE 1 SV 1 | 559.4665 | 927.13 | 311.37 |
| CISY_HUMAN | 15 (13) | 67.62 | 3.5 | Citrate synthase mitochondrial OS Homo sapiens GN CS PE 1 SV 2 | 926.3744 | 1535.61 | 1309.21 |
| TMEDA_HUMAN | 3 | 25.01 | 12.57 | Transmembrane emp24 domain containing protein 10 OS Homo sapiens GN TMED10 PE 1 SV 2 | 44.732 | 74.2 | 266.42 |
| DYN1_HUMAN | 2 | 5.5 | 3.53 | Dynamin 1 OS Homo sapiens GN DNM1 PE 1 SV 2 | 61.9285 | 103.59 | 62.3 |
| ADT3_HUMAN | 54 (8) | 218.29 | 3.53 | ADP ATP translocase 3 OS Homo sapiens GN SLC25A6 PE 1 SV 4 | 391.1729 | 654.69 | 360.77 |
| ATPB_HUMAN | 66 (62) | 416.53 | 3.53 | ATP synthase subunit beta mitochondrial OS Homo sapiens GN ATP5B PE 1 SV 3 | 6017.826 | 10100 | 3369.92 |
| AL3A2_HUMAN | 3 | 13.11 | 3.57 | Fatty aldehyde dehydrogenase OS Homo sapiens GN ALDH3A2 PE 1 SV 1 | 88.6622 | 150.05 | 80.4 |
| POTEI_HUMAN | 43 (1) | 135.13 | 4.37 | POTE ankyrin domain family member I OS Homo sapiens GN POTEI PE 3 SV 1 | 73.7234 | 125.89 | 152.86 |
| TBA4B_HUMAN | 40 (2) | 166.85 | 3.6 | Putative tubulin like protein alpha 4B OS Homo sapiens GN TUBA4B PE 5 SV 2 | 136.1794 | 232.54 | 119.66 |
| TBB8_HUMAN | 70 (3) | 323.83 | 3.61 | Tubulin beta 8 chain OS Homo sapiens GN TUBB8 PE 1 SV 2 | 299.3035 | 512.31 | 269.23 |
| FKB10_HUMAN | 4 | 19.94 | 158.9 | Peptidyl prolyl cis trans isomerase FKBP10 OS Homo sapiens GN FKBP10 PE 1 SV 1 | 5.5071 | 9.46 | 415.4 |
| K6PP_HUMAN | 10 (6) | 45.13 | 3.67 | 6 phosphofructokinase type C OS Homo sapiens GN PFKP PE 1 SV 2 | 179.4555 | 312.13 | 124.73 |
| H2A3_HUMAN | 91 (2) | 289.54 | 3.67 | Histone H2A type 3 OS Homo sapiens GN HIST3H2A PE 1 SV 3 | 71.2125 | 123.93 | 90.75 |
| FUMH_HUMAN | 3 (2) | 18.57 | 3.68 | Fumarate hydratase mitochondrial OS Homo sapiens GN FH PE 1 SV 3 | 75.96 | 132.35 | 59.98 |
| HSPB1_HUMAN | 41 (39) | 227.63 | 31.68 | Heat shock protein beta 1 OS Homo sapiens GN HSPB1 PE 1 SV 2 | 599.24 | 1054.55 | 8996.3 |
| HMGB2_HUMAN | 4 (1) | 24.52 | 3.74 | High mobility group protein B2 OS Homo sapiens GN HMGB2 PE 1 SV 2 | 33.8233 | 59.93 | 22.81 |
| RS25_HUMAN | 10 | 35.26 | 31.97 | 40S ribosomal protein S25 OS Homo sapiens GN RPS25 PE 1 SV 1 | 43.8036 | 78.04 | 663.64 |
| MDHM_HUMAN | 39 (38) | 251.85 | 3.77 | Malate dehydrogenase mitochondrial OS Homo sapiens GN MDH2 PE 1 SV 3 | 2357.334 | 4216.36 | 3821.97 |
| GANAB_HUMAN | 45 (44) | 243.19 | 8.04 | Neutral alpha glucosidase AB OS Homo sapiens GN GANAB PE 1 SV 3 | 1135.855 | 2033.8 | 4325.42 |
| MARCS_HUMAN | 6 | 31.68 | 3.78 | Myristoylated alanine rich C kinase substrate OS Homo sapiens GN MARCKS PE 1 SV 4 | 135.3354 | 242.39 | 86.03 |
| DPYL1_HUMAN | 12 (5) | 75.97 | 3.81 | Dihydropyrimidinase related protein 1 OS Homo sapiens GN CRMP1 PE 1 SV 1 | 199.5638 | 360.45 | 109.94 |
| ARF1_HUMAN | 9 (2) | 31.54 | 29.85 | ADP ribosylation factor 1 OS Homo sapiens GN ARF1 PE 1 SV 2 | 12.7022 | 22.97 | 179.73 |
| ENOG_HUMAN | 108 (34) | 600.42 | 7.37 | Gamma enolase OS Homo sapiens GN ENO2 PE 1 SV 3 | 2501.975 | 4535.43 | 615.14 |
| TPM4_HUMAN | 23 (7) | 155.1 | 9.96 | Tropomyosin alpha 4 chain OS Homo sapiens GN TPM4 PE 1 SV 3 | 131.1576 | 239.49 | 619.15 |
| ROA2_HUMAN | 42 (39) | 256.89 | 5.4 | Heterogeneous nuclear ribonucleoproteins A2 B1 OS Homo sapiens GN HNRNPA2B1 PE 1 SV 2 | 1460.626 | 2687.93 | 3739.9 |
| HNRPM_HUMAN | 3 (2) | 18.15 | 3.88 | Heterogeneous nuclear ribonucleoprotein M OS Homo sapiens GN HNRNPM PE 1 SV 3 | 170.7412 | 314.25 | 135.42 |
| POTEE_HUMAN | 77 (2) | 370.08 | 3.89 | POTE ankyrin domain family member E OS Homo sapiens GN POTEE PE 1 SV 3 | 108.9182 | 200.76 | 99.94 |
| STMN1_HUMAN | 5 (4) | 29.23 | 7.14 | Stathmin OS Homo sapiens GN STMN1 PE 1 SV 3 | 109.2347 | 201.6 | 369.41 |
| GSTP1_HUMAN | 9 | 58.58 | 3.98 | Glutathione S transferase P OS Homo sapiens GN GSTP1 PE 1 SV 2 | 598.2694 | 1127.45 | 717.4 |
| ATPA_HUMAN | 48 (46) | 287.07 | 3.98 | ATP synthase subunit alpha mitochondrial OS Homo sapiens GN ATP5A1 PE 1 SV 1 | 5176.505 | 9767.42 | 3635.89 |
| K1C10_HUMAN | 64 (42) | 310.91 | 4.01 | Keratin type I cytoskeletal 10 OS Homo sapiens GN KRT10 PE 1 SV 6 | 2071.387 | 3933.27 | 1080.36 |
| CBR1_HUMAN | 2 | 12.99 | 13.02 | Carbonyl reductase NADPH 1 OS Homo sapiens GN CBR1 PE 1 SV 3 | 15.2553 | 29 | 94.21 |
| VAT1L_HUMAN | 6 | 39.13 | 4.06 | Synaptic vesicle membrane protein VAT 1 homolog like OS Homo sapiens GN VAT1L PE 1 SV 2 | 194.4998 | 373.79 | 114.91 |
| DPYL2_HUMAN | 27 (21) | 162.97 | 4.08 | Dihydropyrimidinase related protein 2 OS Homo sapiens GN DPYSL2 PE 1 SV 1 | 1471.915 | 2843.51 | 1026.79 |
| PP1B_HUMAN | 3 | 19.2 | 7.27 | Serine threonine protein phosphatase PP1 beta catalytic subunit OS Homo sapiens GN PPP1CB PE 1 SV 3 | 43.9513 | 84.97 | 151.54 |
| ANXA1_HUMAN | 40 (39) | 258.64 | 18.51 | Annexin A1 OS Homo sapiens GN ANXA1 PE 1 SV 2 | 584.4278 | 1145.81 | 5127.53 |
| 1433Z_HUMAN | 48 (30) | 233.76 | 4.25 | 14 3 3 protein zeta delta OS Homo sapiens GN YWHAZ PE 1 SV 1 | 1148.304 | 2253.89 | 2310.52 |
| NDKA_HUMAN | 21 (3) | 109.68 | 4.16 | Nucleoside diphosphate kinase A OS Homo sapiens GN NME1 PE 1 SV 1 | 64.8614 | 128.04 | 103.08 |
| K6PL_HUMAN | 5 (2) | 17.15 | 4.2 | 6 phosphofructokinase liver type OS Homo sapiens GN PFKL PE 1 SV 6 | 74.1665 | 147.49 | 86.59 |
| PYGL_HUMAN | 21 (15) | 100.03 | 4.25 | Glycogen phosphorylase liver form OS Homo sapiens GN PYGL PE 1 SV 4 | 643.8454 | 1295.45 | 417.39 |
| RL17_HUMAN | 2 | 11.22 | 7.68 | 60S ribosomal protein L17 OS Homo sapiens GN RPL17 PE 1 SV 3 | 41.2716 | 83.82 | 150.26 |
| F10A1_HUMAN | 2 | 13.89 | 4.31 | Hsc70 interacting protein OS Homo sapiens GN ST13 PE 1 SV 2 | 78.9351 | 161.31 | 77.9 |
| CRYM_HUMAN | 16 | 93.15 | 4.35 | Thiomorpholine carboxylate dehydrogenase OS Homo sapiens GN CRYM PE 1 SV 1 | 2193.324 | 4525.33 | 1194.95 |
| HNRPK_HUMAN | 20 (19) | 144.37 | 4.38 | Heterogeneous nuclear ribonucleoprotein K OS Homo sapiens GN HNRNPK PE 1 SV 1 | 659.4805 | 1368.16 | 1340.21 |
| ACTN4_HUMAN | 33 (19) | 205.9 | 10.54 | Alpha actinin 4 OS Homo sapiens GN ACTN4 PE 1 SV 2 | 252.6092 | 524.46 | 1261.37 |
| VATA_HUMAN | 10 (9) | 41.99 | 4.43 | V type proton ATPase catalytic subunit A OS Homo sapiens GN ATP6V1A PE 1 SV 2 | 178.9913 | 375.38 | 254.67 |
| DX39B_HUMAN | 2 (1) | 13.35 | 4.71 | Spliceosome RNA helicase DDX39B OS Homo sapiens GN DDX39B PE 1 SV 1 | 50.429 | 106.17 | 112.58 |
| EF1D_HUMAN | 5 (4) | 20.72 | 9.01 | Elongation factor 1 delta OS Homo sapiens GN EEF1D PE 1 SV 5 | 135.6941 | 285.73 | 579.69 |
| KCRB_HUMAN | 89 (88) | 548.74 | 7.27 | Creatine kinase B type OS Homo sapiens GN CKB PE 1 SV 1 | 10161.19 | 21400 | 2939.98 |
| HNRPD_HUMAN | 10 | 63.32 | 4.45 | Heterogeneous nuclear ribonucleoprotein D0 OS Homo sapiens GN HNRNPD PE 1 SV 1 | 736.5377 | 1553.55 | 1015.31 |
| RSSA_HUMAN | 6 | 40.19 | 11.96 | 40S ribosomal protein SA OS Homo sapiens GN RPSA PE 1 SV 4 | 101.6809 | 217.12 | 576.41 |
| COX2_HUMAN | 5 | 17.78 | 4.52 | Cytochrome c oxidase subunit 2 OS Homo sapiens GN MT CO2 PE 1 SV 1 | 206.4846 | 442.24 | 231.41 |
| SC22B_HUMAN | 5 | 28.66 | 8.74 | Vesicle trafficking protein SEC22b OS Homo sapiens GN SEC22B PE 1 SV 4 | 44.5632 | 95.73 | 184.46 |
| TBB1_HUMAN | 33 (3) | 142.28 | 4.55 | Tubulin beta 1 chain OS Homo sapiens GN TUBB1 PE 1 SV 1 | 139.3655 | 300.66 | 96.94 |
| PCBP1_HUMAN | 7 (5) | 42.92 | 7.33 | Poly rC binding protein 1 OS Homo sapiens GN PCBP1 PE 1 SV 2 | 104.0441 | 224.89 | 361.47 |
| GNAO_HUMAN | 16 (11) | 117.55 | 4.59 | Guanine nucleotide binding protein G o subunit alpha OS Homo sapiens GN GNAO1 PE 1 SV 4 | 755.5066 | 1642.82 | 485.97 |
| XRCC5_HUMAN | 2 | 12.11 | 4.6 | X ray repair cross complementing protein 5 OS Homo sapiens GN XRCC5 PE 1 SV 3 | 96.3215 | 209.92 | 158.61 |
| DHE3_HUMAN | 8 | 52.04 | 4.6 | Glutamate dehydrogenase 1 mitochondrial OS Homo sapiens GN GLUD1 PE 1 SV 2 | 232.311 | 506.44 | 317.77 |
| RECO_HUMAN | 23 (21) | 108.85 | 6.88 | Recoverin OS Homo sapiens GN RCVRN PE 1 SV 2 | 2064.677 | 4527.03 | 657.56 |
| CALD1_HUMAN | 3 | 12.49 | 8.28 | Caldesmon OS Homo sapiens GN CALD1 PE 1 SV 3 | 43.2761 | 94.95 | 169.79 |
| LMNB2_HUMAN | 11 (5) | 64.55 | 4.65 | Lamin B2 OS Homo sapiens GN LMNB2 PE 1 SV 3 | 193.2338 | 425.42 | 400.44 |
| PDE6A_HUMAN | 2 | 10.72 | 5.2 | Rod cGMP specific 3 5 cyclic phosphodiesterase subunit alpha OS Homo sapiens GN PDE6A PE 1 SV 4 | 57.7507 | 128.71 | 24.75 |
| NDUS1_HUMAN | 12 | 72.97 | 4.71 | NADH ubiquinone oxidoreductase 75 kDa subunit mitochondrial OS Homo sapiens GN NDUFS1 PE 1 SV 3 | 362.92 | 810.42 | 247.18 |
| AT1A1_HUMAN | 34 (11) | 198.86 | 4.79 | Sodium potassium transporting ATPase subunit alpha 1 OS Homo sapiens GN ATP1A1 PE 1 SV 1 | 351.0618 | 797.58 | 396.96 |
| NUCL_HUMAN | 23 (21) | 118.56 | 6.01 | Nucleolin OS Homo sapiens GN NCL PE 1 SV 3 | 408.496 | 931.58 | 1163.9 |
| RS3A_HUMAN | 7 | 25.8 | 16.26 | 40S ribosomal protein S3a OS Homo sapiens GN RPS3A PE 1 SV 2 | 44.4999 | 101.7 | 343.06 |
| ACTN1_HUMAN | 20 (6) | 127.48 | 17.18 | Alpha actinin 1 OS Homo sapiens GN ACTN1 PE 1 SV 2 | 28.1474 | 64.56 | 229.09 |
| ACTC_HUMAN | 116 (1) | 530.98 | 23.08 | Actin alpha cardiac muscle 1 OS Homo sapiens GN ACTC1 PE 1 SV 1 | 167.4707 | 389.31 | 16.87 |
| CNDP2_HUMAN | 6 | 34.4 | 4.91 | Cytosolic non specific dipeptidase OS Homo sapiens GN CNDP2 PE 1 SV 2 | 198.5299 | 462.34 | 160.21 |
| PSA_HUMAN | 7 | 33.05 | 4.95 | Puromycin sensitive aminopeptidase OS Homo sapiens GN NPEPPS PE 1 SV 2 | 219.44 | 515.08 | 357.95 |
| RL28_HUMAN | 2 (1) | 6.17 | 17.05 | 60S ribosomal protein L28 OS Homo sapiens GN RPL28 PE 1 SV 3 | 4.3044 | 10.12 | 34.77 |
| RS16_HUMAN | 5 | 30.79 | 45.1 | 40S ribosomal protein S16 OS Homo sapiens GN RPS16 PE 1 SV 2 | 24.5604 | 58.24 | 525.01 |
| RL7A_HUMAN | 10 (9) | 49.55 | 12.08 | 60S ribosomal protein L7a OS Homo sapiens GN RPL7A PE 1 SV 2 | 169.5174 | 414.64 | 970.8 |
| RL23_HUMAN | 6 (5) | 26.23 | 14.23 | 60S ribosomal protein L23 OS Homo sapiens GN RPL23 PE 1 SV 1 | 77.7324 | 190.46 | 524.41 |
| HNRPL_HUMAN | 3 | 13.08 | 5.2 | Heterogeneous nuclear ribonucleoprotein L OS Homo sapiens GN HNRNPL PE 1 SV 2 | 73.4702 | 181.14 | 65.87 |
| K2C1B_HUMAN | 10 (1) | 51.07 | 5.22 | Keratin type II cytoskeletal 1b OS Homo sapiens GN KRT77 PE 1 SV 3 | 16.247 | 40.24 | 12.56 |
| VATB2_HUMAN | 6 | 39.96 | 5.29 | V type proton ATPase subunit B brain isoform OS Homo sapiens GN ATP6V1B2 PE 1 SV 3 | 297.9531 | 746.62 | 245.19 |
| LDHB_HUMAN | 39 (31) | 148.43 | 5.33 | L lactate dehydrogenase B chain OS Homo sapiens GN LDHB PE 1 SV 2 | 940.0683 | 2375.8 | 1748.73 |
| UBA1_HUMAN | 15 (14) | 86.89 | 5.34 | Ubiquitin like modifier activating enzyme 1 OS Homo sapiens GN UBA1 PE 1 SV 3 | 291.0112 | 735.97 | 593.18 |
| NSF_HUMAN | 21 (20) | 100.22 | 5.34 | Vesicle fusing ATPase OS Homo sapiens GN NSF PE 1 SV 3 | 557.9895 | 1412.85 | 664.37 |
| TBA1C_HUMAN | 209 (2) | 1050.93 | 65.41 | Tubulin alpha 1C chain OS Homo sapiens GN TUBA1C PE 1 SV 1 | 61.3377 | 155.95 | 2.38 |
| RAB7A_HUMAN | 8 | 47.61 | 8.67 | Ras related protein Rab 7a OS Homo sapiens GN RAB7A PE 1 SV 1 | 116.8729 | 297.33 | 480.29 |
| AT1A3_HUMAN | 62 (35) | 358.09 | 5.38 | Sodium potassium transporting ATPase subunit alpha 3 OS Homo sapiens GN ATP1A3 PE 1 SV 3 | 1577.098 | 4022.79 | 763.27 |
| SRSF3_HUMAN | 6 | 37.19 | 7.93 | Serine arginine rich splicing factor 3 OS Homo sapiens GN SRSF3 PE 1 SV 1 | 130.4824 | 334.33 | 490.57 |
| TERA_HUMAN | 14 (13) | 84.11 | 5.61 | Transitional endoplasmic reticulum ATPase OS Homo sapiens GN VCP PE 1 SV 4 | 236.0035 | 605.84 | 627.16 |
| 4F2_HUMAN | 3 | 19.54 | 5.47 | 4F2 cell surface antigen heavy chain OS Homo sapiens GN SLC3A2 PE 1 SV 3 | 70.0942 | 181.79 | 142.13 |
| UCHL1_HUMAN | 8 (7) | 38.86 | 12.62 | Ubiquitin carboxyl terminal hydrolase isozyme L1 OS Homo sapiens GN UCHL1 PE 1 SV 2 | 90.8144 | 238.06 | 543.06 |
| VDAC1_HUMAN | 34 (33) | 209.12 | 5.63 | Voltage dependent anion selective channel protein 1 OS Homo sapiens GN VDAC1 PE 1 SV 2 | 2044.527 | 5456.22 | 1337.7 |
| NCAM1_HUMAN | 16 | 86.07 | 7.64 | Neural cell adhesion molecule 1 OS Homo sapiens GN NCAM1 PE 1 SV 3 | 1005.415 | 2774.13 | 363.13 |
| APEX1_HUMAN | 2 | 12.4 | 5.83 | DNA apurinic or apyrimidinic site lyase OS Homo sapiens GN APEX1 PE 1 SV 2 | 72.7317 | 201.09 | 59.79 |
| AT1B1_HUMAN | 2 | 13.59 | 10.95 | Sodium potassium transporting ATPase subunit beta 1 OS Homo sapiens GN ATP1B1 PE 1 SV 1 | 94.6968 | 261.92 | 23.91 |
| TALDO_HUMAN | 21 (19) | 129.81 | 5.87 | Transaldolase OS Homo sapiens GN TALDO1 PE 1 SV 2 | 956.0832 | 2661.12 | 930.18 |
| AATC_HUMAN | 6 | 41.34 | 5.88 | Aspartate aminotransferase cytoplasmic OS Homo sapiens GN GOT1 PE 1 SV 3 | 145.5478 | 405.62 | 253.54 |
| AL9A1_HUMAN | 7 | 34.4 | 5.92 | 4 trimethylaminobutyraldehyde dehydrogenase OS Homo sapiens GN ALDH9A1 PE 1 SV 3 | 169.1376 | 474.93 | 282.74 |
| RLA2_HUMAN | 6 | 58.15 | 45.84 | 60S acidic ribosomal protein P2 OS Homo sapiens GN RPLP2 PE 1 SV 1 | 36.9039 | 103.87 | 801.83 |
| PRDX3_HUMAN | 6 (5) | 38.27 | 5.95 | Thioredoxin dependent peroxide reductase mitochondrial OS Homo sapiens GN PRDX3 PE 1 SV 3 | 91.7217 | 258.61 | 185.81 |
| RL22_HUMAN | 4 | 24.46 | 31.66 | 60S ribosomal protein L22 OS Homo sapiens GN RPL22 PE 1 SV 2 | 27.3245 | 77.17 | 410.05 |
| RAN_HUMAN | 11 | 58.26 | 10.54 | GTP binding nuclear protein Ran OS Homo sapiens GN RAN PE 1 SV 3 | 200.9353 | 567.93 | 1003.72 |
| RAB2A_HUMAN | 7 (6) | 36.8 | 12.89 | Ras related protein Rab 2A OS Homo sapiens GN RAB2A PE 1 SV 1 | 68.4273 | 195.22 | 418.17 |
| HXK1_HUMAN | 44 (43) | 204.2 | 6.04 | Hexokinase 1 OS Homo sapiens GN HK1 PE 1 SV 3 | 1914.867 | 5481.91 | 1618.02 |
| NFL_HUMAN | 18 (1) | 38.27 | 25.3 | Neurofilament light polypeptide OS Homo sapiens GN NEFL PE 1 SV 3 | 11.4784 | 32.96 | 137.52 |
| HNRH1_HUMAN | 4 | 26.07 | 6.15 | Heterogeneous nuclear ribonucleoprotein H OS Homo sapiens GN HNRNPH1 PE 1 SV 4 | 90.8144 | 264.65 | 215.86 |
| MPCP_HUMAN | 7 (5) | 30.37 | 6.15 | Phosphate carrier protein mitochondrial OS Homo sapiens GN SLC25A3 PE 1 SV 2 | 165.5084 | 482.76 | 397.32 |
| GABT_HUMAN | 3 (2) | 18.93 | 6.2 | 4 aminobutyrate aminotransferase mitochondrial OS Homo sapiens GN ABAT PE 1 SV 3 | 26.6704 | 78.42 | 43.06 |
| SODM_HUMAN | 4 | 18.19 | 6.21 | Superoxide dismutase Mn mitochondrial OS Homo sapiens GN SOD2 PE 1 SV 2 | 77.3737 | 227.69 | 60.79 |
| ILF2_HUMAN | 4 (3) | 20.55 | 6.26 | Interleukin enhancer binding factor 2 OS Homo sapiens GN ILF2 PE 1 SV 2 | 118.4132 | 351.59 | 172.78 |
| GLNA_HUMAN | 25 | 156.95 | 6.41 | Glutamine synthetase OS Homo sapiens GN GLUL PE 1 SV 4 | 1218.166 | 3698.8 | 1319.74 |
| IMPG1_HUMAN | 8 | 37.56 | 9.81 | Interphotoreceptor matrix proteoglycan 1 OS Homo sapiens GN IMPG1 PE 1 SV 2 | 197.7914 | 602.95 | 61.46 |
| RAB3B_HUMAN | 2 (1) | 11.67 | 6.44 | Ras related protein Rab 3B OS Homo sapiens GN RAB3B PE 1 SV 2 | 10.5922 | 32.34 | 7.17 |
| 1433G_HUMAN | 24 (4) | 111.26 | 6.89 | 14 3 3 protein gamma OS Homo sapiens GN YWHAG PE 1 SV 2 | 57.603 | 176.06 | 188.17 |
| HNRPU_HUMAN | 22 (21) | 127.41 | 6.62 | Heterogeneous nuclear ribonucleoprotein U OS Homo sapiens GN HNRNPU PE 1 SV 6 | 695.9413 | 2182.1 | 1732.49 |
| GNAS1_HUMAN | 5 (2) | 32.51 | 14.11 | Guanine nucleotide binding protein G s subunit alpha isoforms XLas OS Homo sapiens GN GNAS PE 1 SV | 45.5127 | 145.85 | 10.33 |
| THY1_HUMAN | 3 | 20.58 | 30.71 | Thy 1 membrane glycoprotein OS Homo sapiens GN THY1 PE 1 SV 2 | 34.182 | 110.58 | 497.33 |
| HNRPR_HUMAN | 10 (6) | 69.72 | 6.84 | Heterogeneous nuclear ribonucleoprotein R OS Homo sapiens GN HNRNPR PE 1 SV 1 | 104.1074 | 337.26 | 190.76 |
| RLBP1_HUMAN | 35 | 201.56 | 8.5 | Retinaldehyde binding protein 1 OS Homo sapiens GN RLBP1 PE 1 SV 2 | 1406.336 | 4596.06 | 540.99 |
| IMB1_HUMAN | 5 | 30.75 | 42.92 | Importin subunit beta 1 OS Homo sapiens GN KPNB1 PE 1 SV 2 | 16.3103 | 53.57 | 331.58 |
| OPSD_HUMAN | 31 (28) | 162.03 | 20.45 | Rhodopsin OS Homo sapiens GN RHO PE 1 SV 1 | 1658.777 | 5490.93 | 268.49 |
| RS14_HUMAN | 5 | 18.74 | 26.49 | 40S ribosomal protein S14 OS Homo sapiens GN RPS14 PE 1 SV 3 | 25.9108 | 86.25 | 325.36 |
| HP1B3_HUMAN | 3 | 18.29 | 7.03 | Heterochromatin protein 1 binding protein 3 OS Homo sapiens GN HP1BP3 PE 1 SV 1 | 62.2239 | 207.42 | 108.71 |
| SFPQ_HUMAN | 7 | 35.47 | 7.58 | Splicing factor proline and glutamine rich OS Homo sapiens GN SFPQ PE 1 SV 2 | 160.2123 | 540.17 | 575.63 |
| RS19_HUMAN | 4 | 23.15 | 48.67 | 40S ribosomal protein S19 OS Homo sapiens GN RPS19 PE 1 SV 2 | 12.7866 | 43.54 | 294.96 |
| ACON_HUMAN | 16 (15) | 89.79 | 7.22 | Aconitate hydratase mitochondrial OS Homo sapiens GN ACO2 PE 1 SV 2 | 278.9631 | 954.5 | 183.07 |
| PHB2_HUMAN | 18 (16) | 91.46 | 7.51 | Prohibitin 2 OS Homo sapiens GN PHB2 PE 1 SV 2 | 211.2743 | 725.11 | 752.37 |
| ARF4_HUMAN | 10 (2) | 32.5 | 34.58 | ADP ribosylation factor 4 OS Homo sapiens GN ARF4 PE 1 SV 3 | 9.6427 | 33.12 | 158.02 |
| TBB4A_HUMAN | 123 (3) | 635.62 | 7.37 | Tubulin beta 4A chain OS Homo sapiens GN TUBB4A PE 1 SV 2 | 79.4626 | 277.62 | 54.57 |
| PARK7_HUMAN | 28 | 134.46 | 7.48 | Protein DJ 1 OS Homo sapiens GN PARK7 PE 1 SV 2 | 487.5366 | 1728.05 | 624.04 |
| ARRC_HUMAN | 2 | 12.26 | 7.56 | Arrestin C OS Homo sapiens GN ARR3 PE 1 SV 2 | 90.1392 | 322.73 | 60.25 |
| ATPO_HUMAN | 14 (13) | 83.42 | 7.7 | ATP synthase subunit O mitochondrial OS Homo sapiens GN ATP5O PE 1 SV 1 | 308.3132 | 1125.44 | 415.66 |
| GDIA_HUMAN | 6 (3) | 42.15 | 7.74 | Rab GDP dissociation inhibitor alpha OS Homo sapiens GN GDI1 PE 1 SV 2 | 71.1492 | 260.91 | 85.46 |
| SEPT2_HUMAN | 2 | 13.3 | 11.79 | Septin 2 OS Homo sapiens GN SEPT2 PE 1 SV 1 | 24.687 | 91.15 | 7.73 |
| RS8_HUMAN | 8 (7) | 32.77 | 24.02 | 40S ribosomal protein S8 OS Homo sapiens GN RPS8 PE 1 SV 2 | 31.8399 | 118.04 | 362.54 |
| HNRPC_HUMAN | 7 | 48.16 | 7.83 | Heterogeneous nuclear ribonucleoproteins C1 C2 OS Homo sapiens GN HNRNPC PE 1 SV 4 | 164.2002 | 609.1 | 367.6 |
| K6PF_HUMAN | 14 (7) | 52.17 | 7.94 | 6 phosphofructokinase muscle type OS Homo sapiens GN PFKM PE 1 SV 2 | 56.6113 | 213.03 | 120 |
| EFTU_HUMAN | 7 | 39.5 | 7.98 | Elongation factor Tu mitochondrial OS Homo sapiens GN TUFM PE 1 SV 2 | 107.2935 | 405.9 | 196.2 |
| RL27_HUMAN | 4 | 23.49 | 52.23 | 60S ribosomal protein L27 OS Homo sapiens GN RPL27 PE 1 SV 2 | 12.5756 | 48.34 | 311.26 |
| SRSF1_HUMAN | 3 | 11.67 | 8.14 | Serine arginine rich splicing factor 1 OS Homo sapiens GN SRSF1 PE 1 SV 2 | 81.3827 | 314.01 | 95.43 |
| ARRS_HUMAN | 71 (70) | 484.68 | 8.24 | S arrestin OS Homo sapiens GN SAG PE 2 SV 3 | 4588.533 | 17900 | 2616.12 |
| SYPH_HUMAN | 2 | 14.11 | 8.34 | Synaptophysin OS Homo sapiens GN SYP PE 1 SV 3 | 75.0527 | 296.86 | 67.95 |
| STXB1_HUMAN | 10 (8) | 59.81 | 8.43 | Syntaxin binding protein 1 OS Homo sapiens GN STXBP1 PE 1 SV 1 | 203.193 | 812.03 | 304.8 |
| AATM_HUMAN | 15 (14) | 111.23 | 8.45 | Aspartate aminotransferase mitochondrial OS Homo sapiens GN GOT2 PE 1 SV 3 | 585.1663 | 2342.69 | 785.73 |
| S10AB_HUMAN | 6 | 37.98 | 30.77 | Protein S100 A11 OS Homo sapiens GN S100A11 PE 1 SV 2 | 27.43 | 110.42 | 400.06 |
| TBB3_HUMAN | 102 (7) | 562.3 | 11.32 | Tubulin beta 3 chain OS Homo sapiens GN TUBB3 PE 1 SV 2 | 143.9442 | 583.37 | 51.55 |
| PRDX6_HUMAN | 24 (23) | 122.24 | 8.57 | Peroxiredoxin 6 OS Homo sapiens GN PRDX6 PE 1 SV 3 | 531.5301 | 2158.37 | 1205.58 |
| IF5A1_HUMAN | 10 (9) | 32.88 | 50.79 | Eukaryotic translation initiation factor 5A 1 OS Homo sapiens GN EIF5A PE 1 SV 2 | 20.467 | 83.77 | 492.77 |
| VDAC3_HUMAN | 7 (6) | 40.82 | 8.68 | Voltage dependent anion selective channel protein 3 OS Homo sapiens GN VDAC3 PE 1 SV 1 | 91.4685 | 376.09 | 97.51 |
| GNAT1_HUMAN | 25 (20) | 174.53 | 14.86 | Guanine nucleotide binding protein G t subunit alpha 1 OS Homo sapiens GN GNAT1 PE 1 SV 5 | 862.8423 | 3548.14 | 238.7 |
| VDAC2_HUMAN | 21 (20) | 129.92 | 8.71 | Voltage dependent anion selective channel protein 2 OS Homo sapiens GN VDAC2 PE 1 SV 2 | 561.0912 | 2316.13 | 408.31 |
| SPRC_HUMAN | 3 | 20.26 | 100.82 | SPARC OS Homo sapiens GN SPARC PE 1 SV 1 | 3.3549 | 14 | 160.41 |
| XLRS1_HUMAN | 4 | 17.35 | 8.84 | Retinoschisin OS Homo sapiens GN RS1 PE 1 SV 2 | 47.0108 | 197.02 | 93.03 |
| RS3_HUMAN | 12 (11) | 70.55 | 21.3 | 40S ribosomal protein S3 OS Homo sapiens GN RPS3 PE 1 SV 2 | 99.9718 | 421.22 | 1009.32 |
| ALDH2_HUMAN | 2 | 13.58 | 8.9 | Aldehyde dehydrogenase mitochondrial OS Homo sapiens GN ALDH2 PE 1 SV 2 | 28.907 | 121.93 | 24.66 |
| RL34_HUMAN | 3 | 11.52 | 30.1 | 60S ribosomal protein L34 OS Homo sapiens GN RPL34 PE 1 SV 3 | 11.6261 | 49.19 | 165.96 |
| ARPC4_HUMAN | 2 | 11.97 | 27.57 | Actin related protein 2 3 complex subunit 4 OS Homo sapiens GN ARPC4 PE 1 SV 3 | 13.6095 | 57.63 | 177.76 |
| 1433B_HUMAN | 27 (7) | 119.72 | 8.98 | 14 3 3 protein beta alpha OS Homo sapiens GN YWHAB PE 1 SV 3 | 60.9579 | 259.31 | 246.02 |
| RS6_HUMAN | 4 | 25.25 | 40.63 | 40S ribosomal protein S6 OS Homo sapiens GN RPS6 PE 1 SV 1 | 20.7835 | 90.58 | 400.01 |
| MDHC_HUMAN | 12 (11) | 70.8 | 9.22 | Malate dehydrogenase cytoplasmic OS Homo sapiens GN MDH1 PE 1 SV 4 | 323.041 | 1412.15 | 694.73 |
| UCRIL_HUMAN | 2 (1) | 5.86 | 30.66 | Putative cytochrome b c1 complex subunit Rieske like protein 1 OS Homo sapiens GN UQCRFS1P1 PE 5 SV | 6.0979 | 26.94 | 0.88 |
| THIL_HUMAN | 2 | 13.11 | 9.36 | Acetyl CoA acetyltransferase mitochondrial OS Homo sapiens GN ACAT1 PE 1 SV 1 | 21.3321 | 94.67 | 49.49 |
| FSCN1_HUMAN | 5 | 24.32 | 20.94 | Fascin OS Homo sapiens GN FSCN1 PE 1 SV 3 | 46.5888 | 210.68 | 462.2 |
| TPM3_HUMAN | 14 (6) | 87.53 | 48.06 | Tropomyosin alpha 3 chain OS Homo sapiens GN TPM3 PE 1 SV 1 | 25.8686 | 118.54 | 589.17 |
| CAZA2_HUMAN | 4 (2) | 19.33 | 9.77 | F actin capping protein subunit alpha 2 OS Homo sapiens GN CAPZA2 PE 1 SV 3 | 33.8866 | 156.83 | 29.82 |
| QCR2_HUMAN | 9 | 54.21 | 9.81 | Cytochrome b c1 complex subunit 2 mitochondrial OS Homo sapiens GN UQCRC2 PE 1 SV 3 | 125.0597 | 581.3 | 269.83 |
| RS13_HUMAN | 12 (10) | 42.18 | 54.15 | 40S ribosomal protein S13 OS Homo sapiens GN RPS13 PE 1 SV 2 | 14.4746 | 69.83 | 371.6 |
| CDC42_HUMAN | 2 | 12.07 | 17.25 | Cell division control protein 42 homolog OS Homo sapiens GN CDC42 PE 1 SV 2 | 9.0308 | 44.66 | 73.88 |
| DHX9_HUMAN | 9 | 49.83 | 10.45 | ATP dependent RNA helicase A OS Homo sapiens GN DHX9 PE 1 SV 4 | 151.6035 | 751.12 | 531.68 |
| ACTH_HUMAN | 116 (2) | 522.01 | 10.47 | Actin gamma enteric smooth muscle OS Homo sapiens GN ACTG2 PE 1 SV 1 | 44.0146 | 218.4 | 80.31 |
| DDAH1_HUMAN | 10 (9) | 64.91 | 10.52 | N G N G dimethylarginine dimethylaminohydrolase 1 OS Homo sapiens GN DDAH1 PE 1 SV 3 | 192.854 | 961.42 | 243.22 |
| ODPB_HUMAN | 5 (4) | 28.39 | 11.17 | Pyruvate dehydrogenase E1 component subunit beta mitochondrial OS Homo sapiens GN PDHB PE 1 SV 3 | 53.6151 | 283.86 | 182.88 |
| IMMT_HUMAN | 2 | 11.4 | 11.23 | Mitochondrial inner membrane protein OS Homo sapiens GN IMMT PE 1 SV 1 | 18.4836 | 98.4 | 27.26 |
| HS12A_HUMAN | 2 | 11.22 | 321.02 | Heat shock 70 kDa protein 12A OS Homo sapiens GN HSPA12A PE 1 SV 2 | 23.1678 | 124.64 | 0.39 |
| ROA3_HUMAN | 3 | 14.33 | 11.44 | Heterogeneous nuclear ribonucleoprotein A3 OS Homo sapiens GN HNRNPA3 PE 1 SV 2 | 69.6089 | 377.48 | 123.69 |
| CLH1_HUMAN | 32 (31) | 179.53 | 11.5 | Clathrin heavy chain 1 OS Homo sapiens GN CLTC PE 1 SV 5 | 390.1601 | 2126.74 | 1366.97 |
| RS7_HUMAN | 4 | 23.53 | 47.74 | 40S ribosomal protein S7 OS Homo sapiens GN RPS7 PE 1 SV 1 | 11.8582 | 66.33 | 268.42 |
| GDE_HUMAN | 4 | 24.81 | 17.53 | Glycogen debranching enzyme OS Homo sapiens GN AGL PE 1 SV 3 | 80.8974 | 462.14 | 26.37 |
| E41L2_HUMAN | 8 | 45.44 | 12.33 | Band 4 1 like protein 2 OS Homo sapiens GN EPB41L2 PE 1 SV 1 | 78.4498 | 458.52 | 75.52 |
| RL7_HUMAN | 8 (7) | 43.58 | 26.82 | 60S ribosomal protein L7 OS Homo sapiens GN RPL7 PE 1 SV 1 | 28.4217 | 167.15 | 361.26 |
| HNRH3_HUMAN | 3 | 19.83 | 12.41 | Heterogeneous nuclear ribonucleoprotein H3 OS Homo sapiens GN HNRNPH3 PE 1 SV 2 | 37.2837 | 219.34 | 114.64 |
| RAB1C_HUMAN | 10 (4) | 62.11 | 12.67 | Putative Ras related protein Rab 1C OS Homo sapiens GN RAB1C PE 5 SV 2 | 22.7247 | 136.52 | 63.64 |
| HSP74_HUMAN | 9 | 46.59 | 12.77 | Heat shock 70 kDa protein 4 OS Homo sapiens GN HSPA4 PE 1 SV 4 | 83.134 | 503.19 | 308.62 |
| GNAI2_HUMAN | 5 (1) | 34.47 | 12.86 | Guanine nucleotide binding protein G i subunit alpha 2 OS Homo sapiens GN GNAI2 PE 1 SV 3 | 11.9004 | 72.57 | 52.88 |
| ELAV1_HUMAN | 2 | 11.74 | 13.02 | ELAV like protein 1 OS Homo sapiens GN ELAVL1 PE 1 SV 2 | 12.1114 | 74.66 | 74.76 |
| ADT2_HUMAN | 48 (8) | 205.46 | 13.03 | ADP ATP translocase 2 OS Homo sapiens GN SLC25A5 PE 1 SV 7 | 118.8352 | 733.99 | 690.01 |
| HMGB1_HUMAN | 9 (6) | 40.78 | 15.34 | High mobility group protein B1 OS Homo sapiens GN HMGB1 PE 1 SV 3 | 39.9423 | 249.39 | 290.44 |
| DCD_HUMAN | 3 | 18.05 | 13.41 | Dermcidin OS Homo sapiens GN DCD PE 1 SV 2 | 33.2325 | 211.08 | 51.42 |
| ROA1_HUMAN | 16 (15) | 110.88 | 18.68 | Heterogeneous nuclear ribonucleoprotein A1 OS Homo sapiens GN HNRNPA1 PE 1 SV 5 | 137.572 | 882 | 1217.56 |
| ERP29_HUMAN | 5 | 39.1 | 57.3 | Endoplasmic reticulum resident protein 29 OS Homo sapiens GN ERP29 PE 1 SV 4 | 18.5891 | 120.6 | 504.79 |
| PLOD3_HUMAN | 7 (5) | 45.97 | 376.95 | Procollagen lysine 2 oxoglutarate 5 dioxygenase 3 OS Homo sapiens GN PLOD3 PE 1 SV 1 | 1.8146 | 11.96 | 322.85 |
| HMGN1_HUMAN | 5 | 18.85 | 18.68 | Non histone chromosomal protein HMG 14 OS Homo sapiens GN HMGN1 PE 1 SV 3 | 41.1239 | 281.13 | 15.05 |
| KAD1_HUMAN | 2 | 12.59 | 14.76 | Adenylate kinase isoenzyme 1 OS Homo sapiens GN AK1 PE 1 SV 3 | 38.4653 | 269.17 | 55.32 |
| ILF3_HUMAN | 5 | 22.1 | 18.66 | Interleukin enhancer binding factor 3 OS Homo sapiens GN ILF3 PE 1 SV 3 | 30.4895 | 213.56 | 269.55 |
| ATPG_HUMAN | 9 | 51.44 | 14.8 | ATP synthase subunit gamma mitochondrial OS Homo sapiens GN ATP5C1 PE 1 SV 1 | 97.3343 | 682.67 | 93.2 |
| RS23_HUMAN | 2 | 11.94 | 57.5 | 40S ribosomal protein S23 OS Homo sapiens GN RPS23 PE 1 SV 3 | 7.8492 | 56.6 | 213.83 |
| IDH3A_HUMAN | 4 | 26.8 | 15.33 | Isocitrate dehydrogenase NAD subunit alpha mitochondrial OS Homo sapiens GN IDH3A PE 1 SV 1 | 56.8012 | 412.59 | 222.26 |
| PCBP2_HUMAN | 8 (5) | 53.21 | 26.74 | Poly rC binding protein 2 OS Homo sapiens GN PCBP2 PE 1 SV 1 | 31.1225 | 228.88 | 394.36 |
| GBB1_HUMAN | 29 (12) | 149.55 | 15.71 | Guanine nucleotide binding protein G I G S G T subunit beta 1 OS Homo sapiens GN GNB1 PE 1 SV 3 | 305.3381 | 2273.19 | 298.64 |
| TXND5_HUMAN | 2 | 6.03 | 185.96 | Thioredoxin domain containing protein 5 OS Homo sapiens GN TXNDC5 PE 1 SV 2 | 0.6963 | 5.19 | 61.07 |
| RL13A_HUMAN | 2 | 11.38 | 51.96 | 60S ribosomal protein L13a OS Homo sapiens GN RPL13A PE 1 SV 2 | 3.3971 | 25.35 | 83.4 |
| H2AY_HUMAN | 13 (12) | 97.52 | 16 | Core histone macro H2A 1 OS Homo sapiens GN H2AFY PE 1 SV 4 | 286.7068 | 2173.43 | 418.58 |
| PAP1L_HUMAN | 2 | 12.32 | 128.22 | Polyadenylate binding protein 1 like OS Homo sapiens GN PABPC1L PE 2 SV 1 | 1.2871 | 9.89 | 78.67 |
| PHB_HUMAN | 8 | 44.35 | 28.68 | Prohibitin OS Homo sapiens GN PHB PE 1 SV 1 | 37.1782 | 296.61 | 505.53 |
| COPA_HUMAN | 4 | 22.75 | 81.08 | Coatomer subunit alpha OS Homo sapiens GN COPA PE 1 SV 2 | 6.0135 | 49.96 | 231.07 |
| RS26_HUMAN | 3 | 12.27 | 113.21 | 40S ribosomal protein S26 OS Homo sapiens GN RPS26 PE 1 SV 3 | 3.6714 | 31.61 | 196.48 |
| ACLY_HUMAN | 4 | 23.79 | 184.49 | ATP citrate synthase OS Homo sapiens GN ACLY PE 1 SV 3 | 1.7091 | 14.74 | 149.68 |
| RS11_HUMAN | 7 (5) | 24.05 | 31.3 | 40S ribosomal protein S11 OS Homo sapiens GN RPS11 PE 1 SV 3 | 10.8665 | 94.71 | 161.2 |
| RL14_HUMAN | 6 | 40.46 | 50.51 | 60S ribosomal protein L14 OS Homo sapiens GN RPL14 PE 1 SV 4 | 17.6396 | 158.2 | 422.13 |
| PGRC1_HUMAN | 4 (3) | 23.8 | 24.54 | Membrane associated progesterone receptor component 1 OS Homo sapiens GN PGRMC1 PE 1 SV 3 | 14.6012 | 135.44 | 169.92 |
| CAND1_HUMAN | 3 | 16.14 | 21.35 | Cullin associated NEDD8 dissociated protein 1 OS Homo sapiens GN CAND1 PE 1 SV 2 | 14.4746 | 146.44 | 95.79 |
| MATR3_HUMAN | 3 | 17.57 | 22.46 | Matrin 3 OS Homo sapiens GN MATR3 PE 1 SV 2 | 22.5559 | 240.09 | 128.03 |
| KGUA_HUMAN | 4 | 24.87 | 30.62 | Guanylate kinase OS Homo sapiens GN GUK1 PE 1 SV 2 | 18.0616 | 211.12 | 6.89 |
| KRT82_HUMAN | 3 | 11.55 | 25.33 | Keratin type II cuticular Hb2 OS Homo sapiens GN KRT82 PE 1 SV 3 | 26.8814 | 322.84 | 122.35 |
| PURA_HUMAN | 2 | 13.09 | 26.35 | Transcriptional activator protein Pur alpha OS Homo sapiens GN PURA PE 1 SV 2 | 10.339 | 129.13 | 11.13 |
| NB5R3_HUMAN | 6 | 28.87 | 28.1 | NADH cytochrome b5 reductase 3 OS Homo sapiens GN CYB5R3 PE 1 SV 3 | 15.2975 | 193.33 | 203.8 |
| GPDM_HUMAN | 3 | 18.85 | 73.28 | Glycerol 3 phosphate dehydrogenase mitochondrial OS Homo sapiens GN GPD2 PE 1 SV 3 | 2.2788 | 31.34 | 79.02 |
| KCRU_HUMAN | 4 | 18.99 | 29.8 | Creatine kinase U type mitochondrial OS Homo sapiens GN CKMT1A PE 1 SV 1 | 23.9696 | 338.63 | 127.65 |
| TPM1_HUMAN | 18 (7) | 103.41 | 153.03 | Tropomyosin alpha 1 chain OS Homo sapiens GN TPM1 PE 1 SV 2 | 9.0941 | 129.92 | 659.36 |
| PSA7_HUMAN | 2 | 10.94 | 68.99 | Proteasome subunit alpha type 7 OS Homo sapiens GN PSMA7 PE 1 SV 1 | 3.6925 | 54.48 | 121.08 |
| ADT1_HUMAN | 46 (6) | 193.31 | 31.67 | ADP ATP translocase 1 OS Homo sapiens GN SLC25A4 PE 1 SV 4 | 28.8015 | 432.49 | 155.97 |
| KRT83_HUMAN | 11 (2) | 46.3 | 33.58 | Keratin type II cuticular Hb3 OS Homo sapiens GN KRT83 PE 1 SV 2 | 10.8454 | 172.6 | 51.34 |
| PRDX4_HUMAN | 10 (3) | 58.18 | 540.04 | Peroxiredoxin 4 OS Homo sapiens GN PRDX4 PE 1 SV 1 | 0.7596 | 12.3 | 196.28 |
| NFM_HUMAN | 16 (1) | 55.45 | 77.39 | Neurofilament medium polypeptide OS Homo sapiens GN NEFM PE 1 SV 3 | 5.064 | 85.5 | 186.09 |
| RS9_HUMAN | 8 (7) | 43.9 | 240.94 | 40S ribosomal protein S9 OS Homo sapiens GN RPS9 PE 1 SV 3 | 4.4521 | 93.29 | 509.44 |
| DHB12_HUMAN | 2 | 11.23 | 45.12 | Estradiol 17 beta dehydrogenase 12 OS Homo sapiens GN HSD17B12 PE 1 SV 2 | 3.1861 | 68.13 | 42.99 |
| H90B2_HUMAN | 35 (2) | 205.04 | 610.12 | Putative heat shock protein HSP 90 beta 2 OS Homo sapiens GN HSP90AB2P PE 1 SV 2 | 0.5486 | 12.93 | 157.86 |
| RL24_HUMAN | 3 | 11.96 | 250.3 | 60S ribosomal protein L24 OS Homo sapiens GN RPL24 PE 1 SV 1 | 2.11 | 49.88 | 250.52 |
| PCNA_HUMAN | 2 | 6.36 | 160.43 | Proliferating cell nuclear antigen OS Homo sapiens GN PCNA PE 1 SV 1 | 2.6586 | 69.4 | 201.97 |
| HPRT_HUMAN | 4 | 23.38 | 69.87 | Hypoxanthine guanine phosphoribosyltransferase OS Homo sapiens GN HPRT1 PE 1 SV 2 | 4.8319 | 160.04 | 13.68 |
| PCBP3_HUMAN | 7 (2) | 36.34 | 82.47 | Poly rC binding protein 3 OS Homo sapiens GN PCBP3 PE 1 SV 2 | 2.1522 | 84.19 | 1.24 |
| RS4X_HUMAN | 6 | 38.25 | 457.96 | 40S ribosomal protein S4 X isoform OS Homo sapiens GN RPS4X PE 1 SV 2 | 1.1816 | 49.95 | 255.44 |
| GSTM2_HUMAN | 4 (3) | 16.65 | 98.56 | Glutathione S transferase Mu 2 OS Homo sapiens GN GSTM2 PE 1 SV 2 | 1.8779 | 87.69 | 63.11 |
| RL13_HUMAN | 4 (3) | 24.38 | 437.91 | 60S ribosomal protein L13 OS Homo sapiens GN RPL13 PE 1 SV 4 | 0.9073 | 44.79 | 190.4 |
| IPYR_HUMAN | 3 | 10.98 | 104.12 | Inorganic pyrophosphatase OS Homo sapiens GN PPA1 PE 1 SV 2 | 4.4099 | 217.92 | 52.68 |
| PARP1_HUMAN | 2 | 10.86 | 129.3 | Poly ADP ribose polymerase 1 OS Homo sapiens GN PARP1 PE 1 SV 4 | 2.321 | 142.52 | 73.23 |
| RTN4_HUMAN | 2 (1) | 6.33 | 143.26 | Reticulon 4 OS Homo sapiens GN RTN4 PE 1 SV 2 | 0.5908 | 40.54 | 6.4 |
| TBA8_HUMAN | 83 (1) | 483.34 | 184.03 | Tubulin alpha 8 chain OS Homo sapiens GN TUBA8 PE 1 SV 1 | 0.9495 | 83.28 | 1.13 |
| M2OM_HUMAN | 6 | 29.9 | 412.46 | Mitochondrial 2 oxoglutarate malate carrier protein OS Homo sapiens GN SLC25A11 PE 1 SV 3 | 0.9495 | 186.83 | 55.58 |
| ROM1_HUMAN | 3 (2) | 20.02 | Infinity | Rod outer segment membrane protein 1 OS Homo sapiens GN ROM1 PE 1 SV 2 | 0 | 62.68 | 0 |
| CAZA1_HUMAN | 2 (1) | 13.16 | Infinity | F actin capping protein subunit alpha 1 OS Homo sapiens GN CAPZA1 PE 1 SV 3 | 0 | 3.92 | 20.68 |
| RL23A_HUMAN | 2 (1) | 11.95 | Infinity | 60S ribosomal protein L23a OS Homo sapiens GN RPL23A PE 1 SV 1 | 0 | 12.46 | 31.72 |
| EF1B_HUMAN | 2 | 11.47 | Infinity | Elongation factor 1 beta OS Homo sapiens GN EEF1B2 PE 1 SV 3 | 0 | 37.56 | 100.54 |
| RL15_HUMAN | 2 | 11.29 | Infinity | 60S ribosomal protein L15 OS Homo sapiens GN RPL15 PE 1 SV 2 | 0 | 6.62 | 70.48 |
| COBA1_HUMAN | 2 | 11.17 | Infinity | Collagen alpha 1 XI chain OS Homo sapiens GN COL11A1 PE 1 SV 4 | 0 | 2.05 | 152.8 |
| K1H2_HUMAN | 4 (1) | 11.07 | Infinity | Keratin type I cuticular Ha2 OS Homo sapiens GN KRT32 PE 1 SV 3 | 0 | 62.21 | 5.85 |
| GSTM3_HUMAN | 2 (1) | 10.84 | Infinity | Glutathione S transferase Mu 3 OS Homo sapiens GN GSTM3 PE 1 SV 3 | 0 | 27.02 | 13.2 |
| VAMP3_HUMAN | 2 (1) | 6.15 | Infinity | Vesicle associated membrane protein 3 OS Homo sapiens GN VAMP3 PE 1 SV 3 | 0 | 48.91 | 0.47 |
| ERLN2_HUMAN | 2 (1) | 6.08 | Infinity | Erlin 2 OS Homo sapiens GN ERLIN2 PE 1 SV 1 | 0 | 49.51 | 2.77 |
