# Supplementary material for: Comparative proteomic analysis of normal and gliotic PVR retina and contribution of Müller glia to this profile
Source: Exp Eye Res. 2018 Dec;177:197–207. doi: 10.1016/j.exer.2018.08.016 (PMC6280037; doi:10.1016/j.exer.2018.08.016)
Supplement: Multimedia component 2 [file mmc2.docx]

| Accession | Protein name | Peptide count | Abundancy (fmol) |
| --- | --- | --- | --- |
| P08670 | Vimentin | 432 | 66700 |
| P62805 | Histone H4 | 158 | 34300 |
| P60709 | Actin cytoplasmic 1 | 191 | 19800 |
| P07355 | Annexin A2 | 63 | 16800 |
| P68431 | Histone H3 1 | 121 | 16400 |
| P04406 | Glyceraldehyde 3 phosphate dehydrogenase | 125 | 16100 |
| P14618 | Pyruvate kinase isozymes M1 M2 | 148 | 15100 |
| P02545 | Prelamin A C | 84 | 14100 |
| P06733 | Alpha enolase | 226 | 14100 |
| P11021 | 78 kDa glucose regulated protein | 70 | 10400 |
| P04792 | Heat shock protein beta 1 | 41 | 8996.3 |
| P23284 | Peptidyl prolyl cis trans isomerase B | 43 | 8690.1 |
| P68104 | Elongation factor 1 alpha 1 | 50 | 8589.95 |
| P04264 | Keratin type II cytoskeletal 1 | 144 | 8529.87 |
| P14136 | Glial fibrillary acidic protein | 217 | 7957.37 |
| P50454 | Serpin H1 | 54 | 7862.46 |
| P14625 | Endoplasmin | 82 | 7801.21 |
| P60174 | Triosephosphate isomerase | 96 | 6457.41 |
| P62937 | Peptidyl prolyl cis trans isomerase A | 43 | 6308.16 |
| P00338 | L lactate dehydrogenase A chain | 69 | 5803.35 |
| P04083 | Annexin A1 | 40 | 5127.53 |
| P35579 | Myosin 9 | 83 | 5083.56 |
| P07900 | Heat shock protein HSP 90 alpha | 76 | 4383.08 |
| Q14697 | Neutral alpha glucosidase AB | 45 | 4325.42 |
| P11142 | Heat shock cognate 71 kDa protein | 87 | 4274.02 |
| P08758 | Annexin A5 | 60 | 4218.54 |
| P30101 | Protein disulfide isomerase A3 | 33 | 4194.06 |
| P0CG48 | Polyubiquitin C | 37 | 3987.6 |
| P07237 | Protein disulfide isomerase | 39 | 3972.13 |
| P40926 | Malate dehydrogenase mitochondrial | 39 | 3821.97 |

Supplementary Table 2. **Highly abundant proteins in Müller glia cell lines as determined by mass spectrometry analysis.** Table shows the top 30 highly abundant proteins in a pool of 4 Müller glial cell preparations as identified by mass spectrometry. Proteins highlighted in grey were identified to be 2-fold or more upregulated in the gliotic retina as compared to the normal retina in the human mass spectrometry analysis.
