# Supplementary material for: Comparative proteomic analysis of normal and gliotic PVR retina and contribution of Müller glia to this profile
Source: Exp Eye Res. 2018 Dec;177:197–207. doi: 10.1016/j.exer.2018.08.016 (PMC6280037; doi:10.1016/j.exer.2018.08.016)
Supplement: Multimedia component 3 [file mmc3.docx]

| **Pathway name** | **Genes** | **Gene identifier** | **All genes** | **Pgenes** |
| --- | --- | --- | --- | --- |
| LKB1 signaling events | 3 | HSP90AA1, ezrin , 14.3.3 sigma (stratifin) | 43 (43) | 0.000118 |
| Cellular responses to stress | 5 | HSP90AA1, HSPA1L, UBC, HIST1H2BO, HIST1H1D | 267 (284) | 0.000278 |
| Regulation of PLK1 Activity at G2/M Transition | 3 | HSP90AA1, UBC, TUBA1A | 81 (81) | 0.000772 |
| Apoptosis | 3 | Stratifin, vimentin, HIST1H1D | 98 (102) | 0.00134 |
| Attenuation phase | 2 | HSP90AA1, HSPA1L | 28 (30) | 0.00179 |
| SRP-dependent cotranslational protein targeting to membrane | 3 | RPS20, RPL36, SSR4 | 109 (164) | 0.00182 |
| Programmed Cell Death | 3 | 14.3.3 Sigma, vimentin, HIST1H1D | 110 (115) | 0.00187 |
| G2/M Transition | 3 | HSP90AA1, UBC, TUBA1A | 115 (116) | 0.00212 |
| HSF1 activation | 2 | HSP90AA1, HSPA1L | 31 (32) | 0.00219 |
| Mitotic G2-G2/M phases | 3 | HSP90AA1, UBC, TUBA1A | 117 (118) | 0.00223 |
| Striated Muscle Contraction | 2 | Desmin, Vimentin | 32 (32) | 0.00233 |
| Class I PI3K signaling events mediated by Akt | 2 | HSP90AA1, stratifin | 35 (37) | 0.00278 |

**Supplementary table 3. Signalling pathway analysis of proteins identified to be upregulated in the gliotic retina as compared to normal retina.** Table shows the top 12 significant gene pathways corresponding to the proteins which were upregulated in the gliotic retina. The column labelled as ‘Number of genes’ indicate the number of genes identified from a given pathway in the specimens investigated. The column labelled as ‘Number of genes in pathway’ indicates the total number of genes that code for proteins of a given pathway. ‘Pgenes’ indicates the P value of the significance of expression of genes identified in the retinal lysates. Pathway analysis was conducted using IMPaLA online software.
