# Supplementary material for: Comparative proteomic analysis of normal and gliotic PVR retina and contribution of Müller glia to this profile
Source: Exp Eye Res. 2018 Dec;177:197–207. doi: 10.1016/j.exer.2018.08.016 (PMC6280037; doi:10.1016/j.exer.2018.08.016)
Supplement: Multimedia component 4 [file mmc4.docx]

**Supplementary table 4. Signalling pathway analysis of proteins identified to be downregulated in the gliotic retina as compared to normal retina.** Table shows the top 12 significant gene pathways corresponding to the proteins which were downregulated in the gliotic retina. The column labelled as ‘Number of genes’ indicate the number of genes identified from a given pathway in the specimens investigated. The column labelled as ‘Number of genes in pathway’ indicates the total number of genes that code for proteins of a given pathway. ‘Pgenes’ indicates the P value of the significance of expression of genes identified in the retinal lysates. Pathway analysis was conducted using IMPaLA online software.

| **pathway name** | **Genes** | **All genes** | **Pgenes** |
| --- | --- | --- | --- |
| Eukaryotic Translation Elongation | 34 | 92 (148) | 1.96E-34 |
| Eukaryotic Translation Termination | 33 | 86 (139) | 4.42E-34 |
| Peptide chain elongation | 33 | 87 (141) | 7.00E-34 |
| Nonsense Mediated Decay (NMD) independent of the Exon Junction Complex (EJC) | 33 | 91 (144) | 4.12E-33 |
| Formation of a pool of free 40S subunits | 33 | 97 (151) | 4.90E-32 |
| SRP-dependent cotranslational protein targeting to membrane | 34 | 109 (164) | 1.61E-31 |
| Nonsense Mediated Decay (NMD) enhanced by the Exon Junction Complex (EJC) | 33 | 103 (159) | 4.82E-31 |
| Nonsense-Mediated Decay (NMD) | 33 | 103 (159) | 4.82E-31 |
| Cytoplasmic Ribosomal Proteins | 31 | 88 (88) | 1.12E-30 |
| L13a-mediated translational silencing of Ceruloplasmin expression | 33 | 107 (169) | 2.02E-30 |
| 3, -UTR-mediated translational regulation | 33 | 107 (169) | 2.02E-30 |
| GTP hydrolysis and joining of the 60S ribosomal subunit | 33 | 108 (170) | 2.85E-30 |

.
